# Integrative analysis of urinary microRNAs for prostate cancer detection: A proof-of-concept study

**DOI:** 10.1016/j.tranon.2026.102745

**Published:** 2026-04-07

**Authors:** Leila Asadi Samani, Saeid Rahmani, Amir Hossein Kashi, Seyed Amir Mohsen Ziaee, Seyed Javad Mowla

**Affiliations:** a Department of Molecular Genetics, Faculty of Biological Sciences, Tarbiat Modares University, Tehran, Iran; b School of Computer Science, Institute for Research in Fundamental Sciences (IPM), Tehran, Iran; c Urology and Nephrology Research Center, Shahid Labbafinejad Medical Center, Shahid Beheshti University, Tehran, Iran

**Keywords:** Multi‑omics, Liquid biopsy, Urinary microRNA, Prostate cancer, Diagnostic biomarker

## Abstract

**Background:**

Liquid biopsies analyzing circulating nucleic acids offer a non‑invasive strategy for early detection, disease monitoring, and precision medicine. Among these, urinary microRNAs (miRNAs) have emerged as robust biomarkers owing to their stability and regulatory effects on gene expression and tumor progression.

**Methods:**

A multi‑omics integrative analysis combining public microarray, bulk RNA‑seq, and single‑cell RNA‑seq datasets was performed to identify miRNAs differentially expressed between prostate cancer (PCa) patients and healthy controls. The diagnostic potential of these candidates was assessed using receiver operating characteristic (ROC) analysis and support vector machine (SVM) modeling. Validation was conducted through quantitative polymerase chain reaction (qPCR) on urine samples from 19 PCa and 7 benign prostatic hyperplasia (BPH) subjects.

**Results:**

miR-23b-3p demonstrated consistent downregulation across transcriptomic datasets derived from urine, serum, and tissue. ROC and SVM analyses indicated strong diagnostic performance. Urinary qPCR validation yielded an area under the curve (AUC) of 0.79 (95% CI: 0.51–1.00), sensitivity of 0.73 (95% CI: 0.39–0.94), and specificity of 0.86 (95% CI: 0.42–1.00).

**Conclusions:**

The findings suggest that miR-23b-3p represents a promising complementary biomarker for non‑invasive PCa diagnosis. Further large‑scale validation integrating transcriptomic and clinical data is warranted to confirm its clinical applicability.

## Highlights

• Bioinformatics analysis identified urinary miRNAs as stable biomarkers.
• qRT-PCR validation confirmed a non-invasive prostate cancer biomarker.
• Integrative workflow supports robust biomarker discovery in urine.
• The approach avoids uncomfortable DRE, enabling easy urine sampling.

## Background

Prostate cancer (PCa) is the second most common cancer in men and the fifth leading cause of cancer death, primarily affecting middle-aged men. However, the incidence and prevalence of prostate cancer vary by region and are likely influenced by genetic predisposition, family history, race/ethnicity, and various risk factors [1,2].

Prostate-specific antigen (PSA) is a widely used biomarker for PCa diagnosis and monitoring. However, PSA has limitations, including low specificity, overdiagnosis of slow-growing cancers, and uncertain mortality benefit. Consequently, many countries avoid national PSA-based screening to prevent unnecessary treatments that may affect quality of life [3,4]. DRE, another diagnostic tool, is essential for assessing gastrointestinal bleeding, rectal diseases, and prostate abnormalities. However, its utilization is limited due to patient anxiety, discomfort, and physician-related issues, such as a lack of confidence and improper documentation [5]. In a study conducted by Jones et al., the sensitivity and specificity of DRE for predicting PCa in symptomatic patients were reported as 28.6% and 90.7%, with positive and negative predictive values of 42.3% and 84.2%, respectively [6].

The standard diagnostic procedure for PCa, transrectal ultrasound (TRUS)-guided biopsy, has notable limitations, including the risk of infections and missed diagnoses. Radiomics has emerged as a promising approach for integrating imaging-derived quantitative features into clinical decision-making by linking image-based characteristics with relevant clinical outcomes. In prostate cancer, radiomics analyses have been applied across a range of imaging modalities, including multiparametric MRI (e.g., T1- and T2-weighted sequences and diffusion-weighted imaging), transrectal ultrasound, conventional and cone-beam CT, and molecular imaging techniques. Among these, PET/CT using tracers such as radiolabeled prostate-specific membrane antigen (PSMA) and 18F-choline has gained particular attention. The associations between radiomics features and clinical endpoints have been investigated using both conventional statistical methods and, more recently, artificial intelligence–based approaches [7]. MRI/TRUS fusion-guided biopsies have appeared as a more accurate alternative, enhancing the detection of clinically significant tumors. However, the widespread adoption of MRI-based diagnostics is hindered by high costs, limited availability, and the need for specialized expertise. Strategies such as biparametric MRI and same-day imaging are being explored to improve accessibility and cost-effectiveness [8]. Systematic ± fusion tissue biopsy, while the gold standard for diagnosis, can miss 21% to 28% of cancers and underestimate 14% to 17% of cases. Repeated biopsies, although not associated with increased infection rates, often lead to greater patient-reported pain and reduced compliance with follow-up procedures [9,10].

There is growing interest in noninvasive biomarkers for PCa detection, particularly circulating nucleic acids obtained through liquid biopsies. These offer advantages such as early cancer detection, comprehensive tumor characterization, and real-time monitoring of disease progression and treatment response. Liquid biopsies can also track tumor evolution, identify resistance mechanisms, and detect minimal residual disease, making them invaluable for personalized medicine [11]. Among body fluids, urine has gained attention for its noninvasive sampling and ability to monitor tumor dynamics. Urinary nucleic acids, including tumor-derived DNA, mRNA, and miRNA, provide insights into tumor heterogeneity and treatment response [12,13].

MicroRNAs (miRNAs), approximately 22 nucleotides in length, are involved in various stages of cancer development and act as oncogenes or tumor suppressors in multiple types of cancer, including PCa. MiRNA-21 is involved in castration resistance and disease progression. Serum miRNAs from the miRNA-200 and miRNA-17 families are associated with PSA responses and improved survival in patients with castration-resistant prostate cancer (CRPC) treated with docetaxel, making them potential biomarkers for treatment response [14]. The miRNA-200 family regulates epithelial-mesenchymal transition (EMT), which contributes to drug resistance and metastasis, while the miRNA-17 family has immune regulatory functions. Both families may play a role in docetaxel resistance.

MicroRNAs are more stable in biological fluids compared to mRNAs, so that they resist RNA-hydrolyzing enzymes and remain stable even after repeated freeze-thaw cycles. Therefore, due to their stability in biological fluids and resistance to diverse storage conditions, miRNAs are potential candidates for minimally invasive diagnostic and prognostic biomarkers for PCa [12,15,16]. However, challenges remain in discovering reliable urinary miRNA biomarkers due to their low concentrations, lack of standardization in normalization methods, and overlap with other urothelial conditions. Techniques such as quantitative real-time PCR (qRT-PCR), microarrays, and next-generation sequencing (NGS) are employed for miRNA analysis. Among these methods, NGS has been demonstrated to provide higher specificity for detecting different isoforms of miRNAs. Nevertheless, qRT-PCR is still considered a method of choice for validating candidate miRNAs [17].

**Scheme 1:**
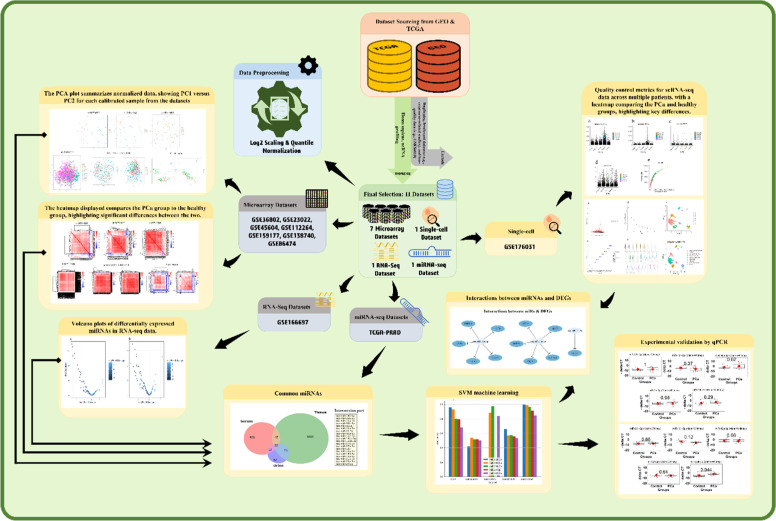
A schematic overview of the study design and workflow for the identification and validation of circulating miRNA biomarkers in prostate cancer.

As outlined in Scheme 1, we analyzed publicly available microarray, RNA sequencing, and single-cell RNA sequencing datasets to identify differentially expressed circulating miRNAs between healthy individuals and patients with PCa. We evaluated the diagnostic potential of these miRNAs using ROC analysis combined with an SVM algorithm. Finally, we validated the most promising miRNA candidate in urine samples using qRT-PCR, to enhance the development of non-invasive biomarkers for prostate cancer diagnosis and screening.

## Materials and methods

### Bioinformatics analysis

#### Dataset selection strategy & microarray analysis workflow

To find urinary miRNAs that are specific to PCa, two main strategies have been employed: screening and validation of PCa-associated miRNAs from urinary microvesicles (exosomes, prostasomes), cell-free miRNAs (cfmiRNAs), or cell pellets, and identification of PCa-associated miRNAs in cell lines or tissues followed by validation in urine samples to enhance specificity and account for contributions from the urinary tract (kidney to bladder) [17]. Datasets were sourced from GEO and TCGA using the query "prostate cancer." Inclusion criteria included "Homo sapiens," "Non-coding RNA profiling by array," and "Non-coding RNA profiling by high-throughput sequencing." After removing duplicates, irrelevant datasets (e.g., treatment-related studies), and low-quality data (e.g., GSE54010), seven microarray datasets (GSE36802, GSE23022, GSE45604, GSE112264, GSE159177, GSE138740, GSE86474), one RNA-seq dataset (GSE166697), one miRNA-seq dataset (TCGA-PRAD), and one single-cell dataset (GSE176031) were selected. All datasets were log2-scaled and quantile-normalized as needed, with R 4.2.0 used as the primary platform for dataset processing and meta-analysis [18]. The seven selected microarray datasets, representing Affymetrix, NanoString nCounter Human miRNA Expression Assay v2, and 3D-Gene Human miRNA platforms, were preprocessed and subjected to quality control. These included GSE36802 (42 tissue samples: 21 PCa vs. 21 benign) [19], GSE23022 (40 tissue samples: 20 tumors vs. 20 adjacent noncancerous tissues) [20], GSE45604 (60 tissue samples: 50 PCa vs. 10 normal) [21], GSE112264 (serum miRNA profiles from 1591 males: 809 PCa, 241 negative PCa, 41 controls, 500 other cancers) [22], GSE159177 and GSE138740 (urinary exosomal miRNA data from 700 males) [23], and GSE86474 (urine miRNA transcriptome data from 152 serial DRE-urine samples)from 10 patients with PCa [24]. Preprocessing steps included data normalization, annotation, and filtering to remove miRNAs with high variability at low intensities, reducing false-positive rates. The quality assessment involved relative log expression (RLE) plots, boxplots of deviations from miRNA medians, hierarchical clustering, principal component analysis (PCA), and heatmap visualizations. Normalization reduced technical variability by adjusting signal intensities across samples [25]. For data from the Affymetrix and 3D-Gene Human miRNA platforms, the normalizeQuantiles function from the Limma package in R was used [26]. For the NanoString platform, the estNormalizationFactors function from the NanoStringDiff package in R was employed, which estimates positive size factors, background noise, and housekeeping factors for the input "NanoStringSet" object [27,28]. Differential expression analyses of miRNAs were conducted using the Limma package for microarray datasets [26] and the NanoStringDiff package for GSE86474(27), with significance thresholds set at *p* < 0.05 and |fold change (FC)| > 1. The Benjamini-Hochberg (B-H) approach was used to handle the false discovery rate (FDR) [29]. For serum datasets including samples from other cancers (e.g., bladder cancer), differential expression analyses were performed to distinguish miRNAs specific to PCa.

#### RNA-seq analysis workflow

Non-coding RNA-seq data from PCa patients were downloaded from the GEO database (accession number GSE166697) [30]. This dataset included 173 urinary extracellular vesicle samples, comprising 46 patients with Gleason Grade ≥ 4 and 127 with Gleason Grade ≤ 3 or biopsy-negative samples. Clinical annotations (PSA levels, DRE status, clinical stage, biopsy results) and standardized sample collection protocols ensured RNA integrity [30]. Raw RNA-seq reads were preprocessed using **TrimGalore** [31] to remove adapter sequences, low-quality reads, low-complexity reads, and contaminants. Sequences shorter than 10 nucleotides or longer than 36 nucleotides were excluded to maintain data integrity. The preprocessed reads were utilizing to the human genome (hg38) using **Bowtie** [32], followed by transcript-level quantification with **HTSeq (v0.9.1)** [33] to count reads mapping to each feature. Differential gene expression analysis was conducted employing **DESeq2** [34] with samples categorized into Gleason high (GG4–GG5) and Gleason low (Bx-negative and GG1–GG3) groups. Significance thresholds were set at adjusted p-values 〈 0.05 and |logFC| 〉 1, with the B-H procedure used to control the FDR.

To complement the RNA-seq findings, miRNA-seq data from TCGA-PRAD (551 tissue samples: 498 tumors vs. 52 normal) [35]were analyzed using **DESeq2** and **edgeR** to identify differentially expressed miRNAs potentially involved in PCa progression [34,36]. This dual-package approach enhances the reliability of the findings and ensures the detection of key miRNAs.

#### Integration of differential expression results

To integrate results from various analyses, the RobustRankAggreg (RRA) method was employed. This method uses a probabilistic model to aggregate ranked lists robustly, even in the presence of noise, by calculating significance probabilities for each element. It is widely recognized for handling diverse gene list aggregation tasks [37].

The RRA package in RStudio, as previously utilized in similar studies [[37], [38], [39]] was employed to integrate and rank miRNAs across multiple datasets. The RRA package in R was used to integrate and rank miRNAs across multiple datasets. miRNAs from each dataset were sorted based on adjusted p-values, and the sorted lists were input into the aggregateRanks () function. This process consolidated the rankings into a unified list, reflecting overall miRNA significance while accounting for noise and variability.

The RRA-generated rankings were used to identify common miRNAs across serum and tissue datasets; subsequently, those shared among urine, serum, and tissue datasets. The intersect () function in R facilitated this process, ensuring a systematic approach to identifying miRNAs of interest for further investigation.

#### Machine learning workflow

MiRNA expression data were obtained from publicly available datasets, including GSE112264, GSE166697, GSE159177, GSE104429, and TCGA-PRAD, covering both PCa and non-cancer samples. Expression matrices were processed using R, with annotations derived from platforms GPL21263, GPL18573, GPL21572, and GPL22634. Transcriptome profiling data from TCGA-PRAD were used for precise miRNA identifier mapping.

Metadata files were utilized to classify samples into "ProstateCancer" and "Norm" groups, excluding samples from other categories to maintain focus and analytical consistency. Samples with missing values were removed to ensure data quality. The expression matrix rows were indexed by miRNA IDs, and low-expression miRNAs were filtered based on median log-transformed counts per million (CPM). miRNAs with a median log2 CPM below −0.2 were excluded to retain biologically relevant features and minimize noise. Normalization was performed using the edgeR package to compute CPM values, ensuring comparability across samples. Both raw and log2-transformed CPM values were retained for downstream analyses. Log2 transformation was used to stabilize variance and decrease the influence of outliers, a critical step in high-throughput expression data analysis. To explore the dataset structure, Principal Component Analysis (PCA) was performed using the prcomp function in R. PCA was applied to the transposed expression matrices, and the first few principal components were visualized in 2D and 3D spaces. A 3D scatter plot was developed utilizing the rgl package [40], with instances color-coded by group ("ProstateCancer" or "Norm"). This visualization helped identify clustering patterns and potential overlaps between the groups. MiRNAs differentially expressed between cancerous and non-cancerous samples were recognized utilizing the limma package. A linear model was fitted to the normalized expression data, with contrast matrices constructed to compare the two groups. The empirical Bayes approach was applied to moderate standard errors, enhancing statistical reliability. miRNAs were ranked based on log-fold changes and adjusted p-values, with FDR correction via the Benjamini-Hochberg procedure. The annotated results were cross-referenced with platform annotation files for further interpretation. To refine the classification model, 20 miRNAs were selected as the intersection of differentially expressed miRNAs across tissue, serum, and urine datasets. From these, 8 miRNAs with the highest biological relevance were chosen for model training. SVM classifiers were trained sequentially with 1 to 8 miRNAs, and performance was estimated utilizing ROC curves and AUC values. The goal was to identify the **smallest miRNA subset** with the best diagnostic performance. This process led to the selection of a final **panel of 5 miRNAs** that demonstrated superior performance. SVM classifier was implemented in R to evaluate the diagnostic potential of the identified miRNAs. The dataset was split into training (60%) and testing (40%) subsets. A kernel-based approach was used to optimize the decision boundary, leveraging SVM’s ability to maximize the margin in high-dimensional space for robust classification. SVM has been widely used in prostate cancer classification studies [[41], [42], [43], [44], [45]].

**Fig. 1:**
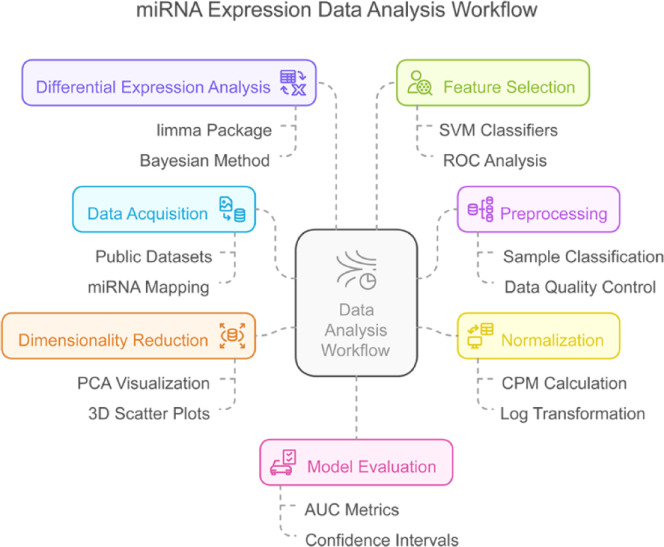
**This diagram explains the entire procedure of extracting and analyzing expression data for miRNA**. The procedure starts from Data Acquisition, in which public datasets are accessed and miRNAs are mapped. Next is Preprocessing, which takes into account sample segregation and quality control checks on the data. In order to make samples adequately comparable through the incorporation of CPM computation and logarithmic transformation, normalization is executed. For data structure and variability exploration, Dimensionality Reduction includes PCA visualization and scatter plotting in three dimensions (3D). The Bayesian approach, along with the limma package and other Bayesian statistical tools, is applied to carry out Differential Expression Analysis. SVM classifiers, along with ROC analysis, are performed to select important biomarkers for Feature Selection. These experiments are analyzed through AUC proportions with accompanying confidence intervals attached to determine the strength and reliability of the model in Model Evaluation.

ROC curve analysis was used to evaluate the model's performance, utilizing the pROC package to compute and visualize the ROC curves [46]. The AUC served as a summary metric of the classifier’s discriminative power, quantifying the likelihood of correctly distinguishing prostate cancer from non-cancer samples. Confidence intervals for sensitivity were also calculated and displayed as shaded regions on the ROC plot. The workflow for processing miRNA data, from acquisition to model evaluation, is illustrated in Fig. 1.

#### Single-cell RNA-seq workflow

Leveraging the high resolution of single-cell RNA sequencing (scRNA-seq), this study aimed to precisely characterize gene expression in cancer cells and explore the relationship between genes and miRNAs. By analyzing single-cell data, we sought to provide deeper insights into their regulatory interactions in PCa.

The scRNA-seq data of PCa patients were obtained from the GEO database (accession number GSE176031) [47]. The dataset included scRNA-seq profiles from 53 PCa specimens and matched benign tissues, derived from biopsies, prostatectomy samples, and patient-derived organoids. For this study, we focused on a subset of 19 prostatectomy samples from five patients. Data preprocessing and quality control were conducted employing the "Seurat" package in R [48]. Cells with fewer than 50 detected genes, genes expressed in fewer than three cells, mitochondrial gene percentages > 25%, or ERCC counts > 25% were excluded. These stringent measures ensured high-quality data for downstream analysis [49]. Normalization was applied utilizing the NormalizeData function to scale gene expression levels within each cell, accounting for sequencing depth differences. This involved dividing each gene’s expression by the total expression in the cell, multiplying by a scale factor of 10,000, and log-transforming the data. The top 2000 most variable genes were selected using the FindVariableGenes function for PCA. Scaling was conducted using the ScaleData function to center and scale expression levels of highly variable genes, mitigating the impact of overexpressed genes (e.g., cell cycle or stress-related genes) through linear regression [49]. Linear dimensionality reduction was conducted using PCA, and the first 20 principal components (PCs) were used for t-SNE and UMAP visualizations via the RunTSNE and RunUMAP functions. The harmony package was used to correct for batch effects, which integrates the dataset while preserving biodiversity. Cells were clustered utilizing the FindClusters function in Seurat, a graph-based approach that constructs a shared nearest neighbor graph using PCA. A resolution of 0.1 was selected after testing a range of values (0.1 to 1). Cell type identification was performed using Seurat’s FindAllMarkers function to detect DEGs across clusters. Marker genes were matched to known cell types based on the literature and single-cell databases [47,50,51].

Main cell populations were annotated utilizing well-characterized marker genes:

• **Endothelial cells:** EMP1, TM4SF1, EMCN
• **Myeloid cells:** IL8, IL1B, HLA-DQA1
• **T-cells:** TRBC2, CCL5, IL7R
• **Mast cells:** KIT, CPA3, TPSAB1
• **Basal epithelial cells:** KRT15, KRT5, SLC14A1
• **Fibroblasts:** DCN, C7, C1S
• **Smooth muscle cells:** SYNPO2, RGS5, LMOD1
• **Luminal epithelial cells:** ANPEP, NIPAL3, ALOX15B
• **Tumor cells:** PCA3, ERG, OR51E2 [47,52].

DEG analysis was performed between cell types to identify genes that were significantly upregulated or downregulated in specific cell populations. The FindMarkers function from the Seurat package was employed for pairwise comparisons, applying a Wilcoxon rank-sum test to evaluate the significance. To correct for multiple testing, p-values were adjusted utilizing the B-H approach to control the FDR. Genes with an adjusted p-value < 0.05 and a log fold change (logFC) > |1| were considered differentially expressed. To explore miRNA regulatory roles, miRNA-target interactions were predicted using **miRDB** [53] and visualized using **Cytoscape 3.9.0** [54], providing insights into miRNA-DEG regulatory networks in PCa.2.6. Patient samples.

### Experimental methods

#### Patient samples

After selecting the miRNA panel through in silico analysis, its diagnostic potential was validated using quantitative polymerase chain reaction (qPCR) to differentiate PCa from BPH. Urine samples were collected from 19 PCa patients and 7 BPH patients at Labbafi Nezhad Hospital, Tehran, Iran. The clinicopathological features of the patients in this study are given in Table 1. Samples were collected during the same session as prostate sampling to ensure consistency. A 10- or 12-French Nelaton catheter was used for urine collection under sterile conditions, minimizing patient discomfort. Catheterization was performed for patients undergoing procedures like biopsy, surgery (OP), or TURP after induction of anesthesia. This allowed for urine collection and minimized discomfort. Trained urology personnel conducted all procedures, reducing the risk of hematuria and ensuring sample quality.

**Table 1:** Summary of clinicopathological features of patients.

Samples		BPH (*n* = 7)	PCa (*n* = 19)
Age, years; mean(range)		71.1(62–81)	66.7(57–88)
Serum PSA concentrations,	≤10 ng/dL	0	9
	>10 ng/dL	7	10
Grade group	I, II	-	7
	III, IV, V	-	12

Due to the inherent limitations of the DRE test, this method was not employed before sample collection. BPH patients were selected based on confirmed benign pathology from open prostatectomy (OP) or transurethral resection of the prostate (TURP). PCa patients underwent radical prostatectomy, with surgical pathology confirming the diagnosis.

All urine specimens were kept at −20 °C until RNA extraction was performed. The ethics of this study were checked and approved by the Ethics Committee of the Urology and Nephrology Research Center (IR.SBMU.UNRC.REC.1401.027, 25 ⁄ 12 ⁄ 2022) and Tarbiat Modares University (Approval Number: IR.MODARES.REC.1401.041). All participants provided verbal informed consent before sample collection.

#### Stem-loop primers design

MiRNAs are small, typically 17 to 24 nucleotides (nt) long, which is shorter than the target sequence required for standard qPCR methods (≥40 nt). To address this, a stem-loop primer was utilized for first-strand cDNA synthesis, extending the miRNA sequence to over 60 nucleotides. This allowed for PCR amplification using a forward primer with an additional five nucleotides to achieve the appropriate melting temperature (Tm) and a universal reverse primer complementary to the RT stem-loop primer [55]. Stem-loop primers were synthesized, cloned into TA vectors (pGEM®-T Vector, A1360), and verified by sequencing to ensure specificity. The primer sequences utilized in this study are detailed in Table S1 of the supplementary file.

#### Extraction of total RNA

Before RNA extraction, 500 μL of urine was incubated at 56 °C for 1 hour with Proteinase K (Molekula, United Kingdom), followed by total RNA extraction using YTzol Pure RNA (Yekta Tajhiz Azma, Iran) according to the manufacturer's protocol. RNA was then quantified using a NanoDrop ND-1000 spectrophotometer (NanoDrop Technologies; Thermo Fisher Scientific, Inc., Wilmington, DE). Typically, a minimum of 200 ng of RNA needs to be applied to a denaturing agarose gel to be seen with ethidium bromide [56]. Due to the lower concentration of RNA obtained from our urine samples, we were unable to evaluate the quality of the extracted RNA using gel electrophoresis.

#### cDNA synthesis

Initially, the extracted RNA was treated with DNase I (Thermo Fisher Scientific, US) at 37 °C for 30 min. Subsequently, cDNA synthesis was performed according to the manufacturer's protocol using the cDNA synthesis kit (Yekta Tajhiz Azma, Iran) with 11 μL of DNase-treated RNA in a total volume of 20 μL.

#### Quantitative real-time PCR

The expression of chosen miRNAs was measured using the stem-loop method with EvaGreen reagent (Ampliqon, Denmark). The mature miRNAs analyzed included:

• miR-331–3p: MIMAT0000760
• miR-191–5p: MIMAT0000440
• miR-92a-3p: MIMAT0000092
• miR-24–3p: MIMAT0000080
• miR-23b-3p: MIMAT0000418

5S rRNA served as the internal control for normalization [57,58]. Reactions were performed in duplicate on an ABI 7500 system (Applied Biosystems, Foster City, CA). The qPCR cycling conditions were: initial denaturation at 95 °C for 10 min, followed by 45 cycles of 95 °C for 15 s, 61 °C for 20 s, and 72 °C for 20 s, with a final melting curve analysis.

#### Statistical analysis

The Mann-Whitney U test was used to analyze the raw expression data for each miRNA in the PCa and control groups, as the data did not follow a normal distribution (Shapiro-Wilk test). Normalized ΔCt values (relative to 5S rRNA) were compared between groups utilizing the Mann-Whitney U test. Graphical data represent mean expression values across two technical replicates for each miRNA. ROC curve analysis was conducted utilizing the pROC package in R to evaluate the diagnostic potential of chosen miRNAs in distinguishing PCa from BPH. The AUC was reported alongside 95% confidence intervals (CI) to indicate the precision and statistical significance of the diagnostic performance. Sensitivity and specificity at the optimal threshold were calculated based on the Youden index, and only numeric values were reported to avoid misleading NAs.To evaluate the effect of age on biomarker performance, a logistic regression model was created using miR-23b-3p ΔCt values as the predictor and age as a covariate through the glm function in R. The predicted probabilities from this model generated age-adjusted ROC curves with AUC and 95% CI to assess how age influences the distinction between PCa and BPH. Additionally, ROC analysis for PSA was conducted using the pROC package in R. Raw PSA values were directly input into the roc function to evaluate their diagnostic capability. To determine the impact of PSA on biomarker performance, a logistic regression model was developed with miR-23b-3p ΔCt values as the predictor and PSA as a covariate using the glm function in R. This generated PSA-adjusted ROC curves with AUC and 95% CI to evaluate how PSA distinguishes between PCa and BPH. Finally, a combined multivariable logistic regression model was developed using the glm function in R, including miR-23b-3p ΔCt, age, and PSA. The predicted probabilities from this model were utilized alongside the roc function from the pROC package to create a composite ROC curve. From this curve, the AUC with 95% CI, optimal threshold, sensitivity, and specificity were calculated and reported, ensuring the diagnostic metrics are interpretable and statistically robust.

## Results

### Bioinformatics results

#### Microarray workflow

**Fig. 2:**
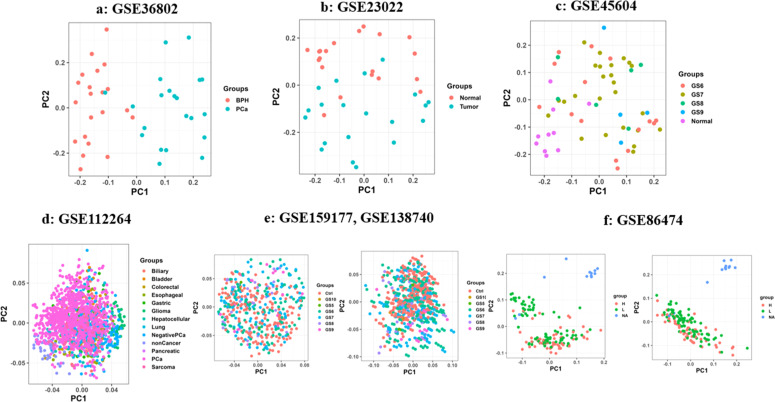
**The PCA plot summarizes normalized data, showing PC1 versus PC2 for each calibrated sample from the datasets.** Note: Figs. 2e and 2f show the PCA plot for documents GSE159177, GSE138740, and GSE86474 before (left) and after (right) batch effect correction.

**Fig. 3:**
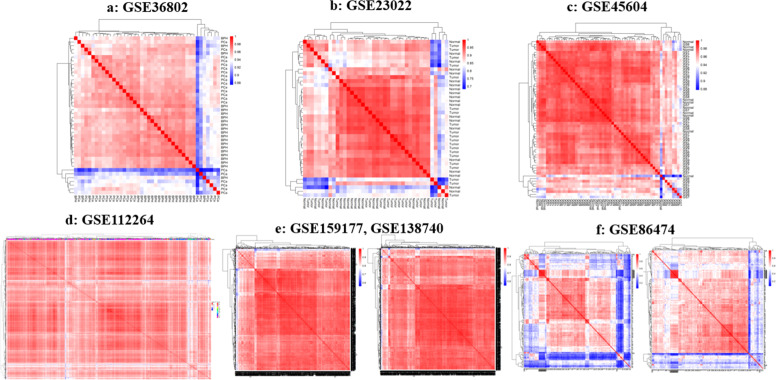
The heatmap displayed compares the PCa group to the healthy group, highlighting significant differences between the two. Note: Figs. 3e and 3f for GSE159177, GSE138740, and GSE86474 show the heatmap results before (left) and after (right) correcting for batch effects.

Seven microarray datasets from the GEO database (GSE36802, GSE23022, GSE45604, GSE112264, GSE159177, GSE138740, GSE86474) were selected, representing Affymetrix, NanoString nCounter Human miRNA Assay v2, and 3D-Gene Human miRNA platforms. These datasets included diverse sample types (serum, urine, tissue) collected under varying conditions. Due to heterogeneity in sample sources and protocols, datasets were analyzed separately, except for GSE159177 and GSE138740, which were combined after batch effect adjustment. Preprocessing included rigorous normalization and filtering to reduce false positives and technical variability. For Affymetrix and 3D-Gene platforms, the normalizeQuantiles function (Limma package) was used, while the estNormalizationFactors function (NanoStringDiff package) was applied to NanoString data. These steps ensured consistent signal intensity distributions across samples. The quality assessment involved generating RLE plots, with post-normalization distributions centered around zero, indicating improved quality (Figure S1, Supplementary file). PCA revealed a clear separation between PCa and control groups in principal component space, with tighter clustering post-batch correction for datasets GSE159177, GSE138740, and GSE86474 (Fig. 2). Heatmaps were generated to compare miRNA expression between PCa and healthy groups, highlighting significant expression differences (Fig. 3). For datasets requiring batch correction (GSE159177, GSE138740, GSE86474), Figs. 3e and 3f demonstrate clearer sample segregation post-correction, confirming the reliability of the preprocessing workflow. The Limma package was used for differential expression analysis of the microarray dataset, and NanoStringDiff was used for GSE86474, with significance thresholds set at an adjusted p-value < 0.05 and |fold change (FC)| > 1, and corrected utilizing the Benjamini-Hochberg method. For datasets containing non-PCa samples (e.g., bladder cancer in serum), specific analyses identified miRNAs uniquely associated with prostate cancer. A complete list of differentially expressed miRNAs (DE-miRNAs) is supplied in Supplementary Tables S2–10.

#### RNA-seq workflow

**Fig. 4:**
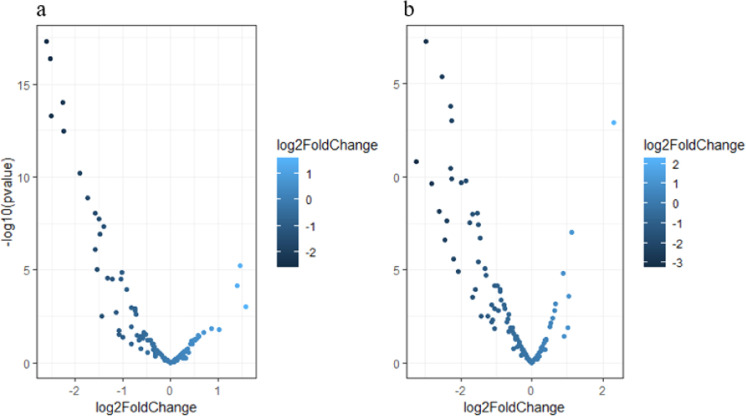
**Volcano plots of differentially expressed miRNAs in RNA-seq data.** (a) Differential miRNA expression between Gleason low (GG1–GG3) – NA groups. (b) Differential miRNA expression between Gleason high (GG4 and GG5) – NA. Each point represents a miRNA, with the x-axis showing the log2 fold change (expression difference between groups) and the y-axis showing the -log10(p-value). MiRNAs with significant differential expression (adjusted p-value 〈 0.05 and |log2FoldChange| 〉 1) are highlighted. The gradient of blue represents the magnitude of the log2 fold change.

This study analyzed urinary extracellular vesicle (EV) samples from PCa patients, with preprocessing and quality control performed using TrimGalore to trim sequences (10–36 nucleotides), remove adapters, and filter low-quality reads. Reads were aligned to the hg38 genome utilizing Bowtie, achieving high mapping efficiency. MiRNA abundances were quantified using htseq-count and featureCounts with GFF/GTF annotations, ensuring a reliable dataset for exploring urinary RNA profiles and their association with PCa. Differential expression analysis using DESeq2 identified significantly DEGs between Gleason high (GG4–GG5) – NA (biopsy-negative) and Gleason low (GG1–GG3) – NA groups. DEGs were chosen based on stringent criteria: adjusted *p*-value 〈 0.05 and |logFC| 〉 1. Volcano plots in Fig. 4 illustrate the distribution of DEGs, highlighting significantly dysregulated miRNAs with clear separation between upregulated and downregulated candidates. Detailed lists of differentially expressed miRNAs for Gleason high–NA versus Gleason low–NA comparisons are provided in Supplementary Tables S11 and S12, identifying potential miRNAs involved in PCa progression. For mir-seq data, DEGs were also determined utilizing both DESeq2 and edgeR, with pairwise comparisons between primary tumors and normal tissues revealing 337 significant DEGs (adjusted p-value 〈 0.05, |logFC| 〉 1) with edgeR and 317 DEGs with DESeq2. Low-expressing miRNAs were filtered using a threshold, as shown by the red line in Figure S2 (supplementary file). The top 30 DEGs identified by each method are listed in Supplementary Tables S13 (edgeR) and S14 (DESeq2), ranked by adjusted p-values.

#### RobustRankAggreg workflow

**Fig. 5:**
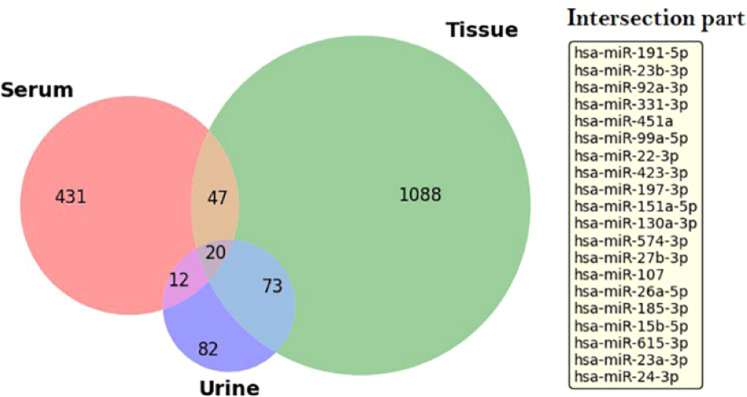
A set of 20 miRNAs was consistently identified across serum, tissue, and urine samples, highlighting their common presence and potential significance as robust, non-invasive biomarkers. The recurrence of these specific miRNAs in multiple biological sources underscores their relevance in prostate cancer detection and monitoring, suggesting they may reflect systemic and localized tumor-associated molecular changes.

Differential miRNA expression results across multiple datasets were integrated using the RRA method, consolidating rankings into a unified list to identify miRNAs consistently significant across serum, tissue, and urine datasets. This approach ensured robust and reliable detection of biologically relevant candidates. The analysis began by identifying 67 common miRNAs between serum and tissue datasets using the **intersect()** function in R (Supplementary Table S15), representing key players in prostate cancer pathogenesis due to their cross-tissue consistency. To identify miRNAs with broader relevance, urine datasets were integrated with serum and tissue data, revealing 20 common miRNAs across all three sources. These miRNAs were ranked using the RRA method, prioritizing candidates based on statistical significance and cross-dataset consistency (Supplementary Table S16), with Fig. 5 visually representing these shared miRNAs. Literature evidence supports their role in prostate cancer progression. From the ranked list of 20 common miRNAs, a subset was selected for further analysis using SVM-based classification, chosen based on strong statistical significance, cross-dataset consistency, and known biological relevance in prostate cancer. These miRNAs will be evaluated in the next section for their potential as diagnostic and prognostic biomarkers.

#### Machine learning workflow

To identify diagnostic biomarkers for prostate cancer (PCa), 20 miRNAs consistently differentially expressed across tissue, serum, and urine datasets were selected. Eight miRNAs, chosen based on biological significance and RRA ranking scores, were used to train an SVM model to distinguish PCa from normal samples. The five best-performing miRNAs were selected to make a final diagnostic panel. Model performance was calculated employing AUC across multiple datasets (TCGA, GSE166697, GSE159177, GSE104429, GSE112264), ensuring consistent classification accuracy across diverse sample types and conditions. Each of the eight miRNAs was evaluated for its ability to classify PCa and normal samples. miR-92a-3p and miR-191–5p showed the highest performance in TCGA, with AUC values of 0.956 and 0.931, respectively. However, their performance varied across datasets; for example, miR-92a-3p achieved an AUC of 0.956 in TCGA but only 0.417 in GSE166697, highlighting dataset-dependent variability. Table 2 summarizes the AUC values for the top five miRNAs across different datasets. AUC values for double and triad combinations of five selected miRNAs are in Supplementary Tables S17 and S18.

**Table 2:** AUC values of miRNAs in databases. NA: The miRNA is not available in this dataset.

miRNA	TCGA	GSE166697	GSE159177	GSE104429	GSE112264
miR-92a-3p	0.956 (0.927–0.985)	0.417 (0.302–0.531)	NA	0.656 (0.535–0.778)	1.000 (1.000–1.000)
miR–191–5p	0.931 (0.899–0.962)	0.536 (0.434–0.638)	0.882 (0.772–0.992)	0.570 (0.497–0.643)	0.985 (0.971–0.999)
miR-23b-3p	0.800 (0.738–0.861)	0.513 (0.487–0.539)	0.973 (0.920–1.000)	0.571 (0.513–0.630)	0.964 (0.944–0.984)
miR–24–3p	0.795 (0.736–0.854)	0.518 (0.483–0.553)	NA	0.557 (0.504–0.611)	0.910 (0.878–0.943)
miR–331–3p	0.682 (0.609–0.754)	0.500 (0.500–0.500)	0.837 (0.724–0.951)	0.530 (0.489–0.572)	0.844 (0.804–0.884)

**Fig. 6:**
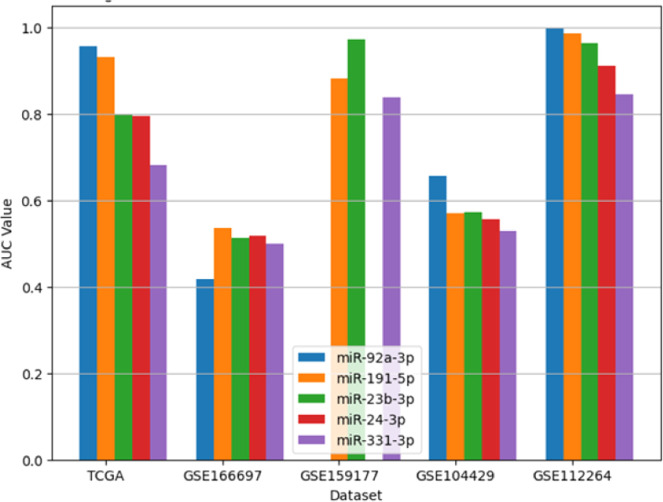
AUC performance of individual miRNAs across datasets.

Due to variability in individual miRNA performance, a refined diagnostic panel was developed using the five miRNAs with the most consistent AUC values across datasets. These miRNAs were selected for their robust accuracy in TCGA tissue samples and reasonable performance in external cohorts. An SVM model trained on this five-miRNA panel demonstrated improved robustness compared to models using individual miRNAs or the initial eight-miRNA set. Fig. 6 shows ROC curves for the panel across datasets, confirming its diagnostic potential. Performance variability likely arises from differences in sample sources (tissue, serum, urine), preprocessing methods, and patient diversity (e.g., DRE procedures, geographical/racial factors). The final panel was chosen for its strong TCGA performance and generalizability across datasets. Future studies will validate the panel using real-time PCR in clinical samples to differentiate PCa from BPH and improve clinical applicability.

#### Single-cell RNA-seq workflow

**Fig. 7:**
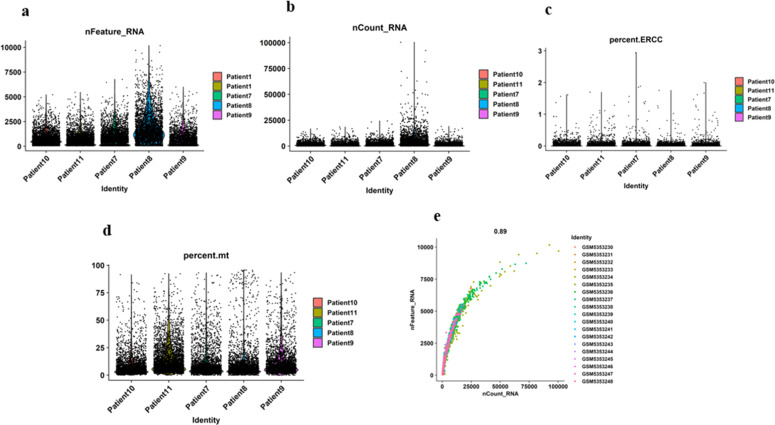
**Quality control metrics for scRNA-seq data across multiple patients**. (a) Number of detected genes (nFeature_RNA) per cell. (b) Total unique molecular identifiers (UMIs) per cell (nCount_RNA).(c) Percentage of ERCC spike-in RNA per cell (percent.ERCC).(d) Percentage of mitochondrial gene expression per cell (percent.mt). (e) Correlation between the number of detected genes and the total UMI counts, with a Pearson correlation coefficient of 0.89.

We utilized scRNA-seq to investigate cellular heterogeneity and gene expression in cancer cells, with a focus on gene-miRNA interactions. The scRNA-seq data from GSE176031 included 19 prostatectomy-derived specimens from five prostate cancer patients (patients 7–11), filtered from an initial pool of 53. Figs. 7a–e display sample statistics, including total genes expressed, mitochondrial and ERCC sequencing counts, and sequencing depth correlations. Fig. 7d shows low mitochondrial percentages, likely due to minimal apoptotic or lysed cells during sorting. Fig. 7e illustrates the correlation between sequenced genes and sequencing depth. After stringent QC filtering, 20,488 high-quality cells were retained. Raw read counts were normalized, scaled, and regressed against gene count expression, with the 2000 most variable features selected for downstream analysis (Fig. 7a) [59]. PCA was used on the scaled data to determine the optimal number of PCs that capture the dataset's variance. Elbow plots in Fig. 7c aided in selecting the appropriate number of PCs for downstream analysis.

**Fig. 8:**
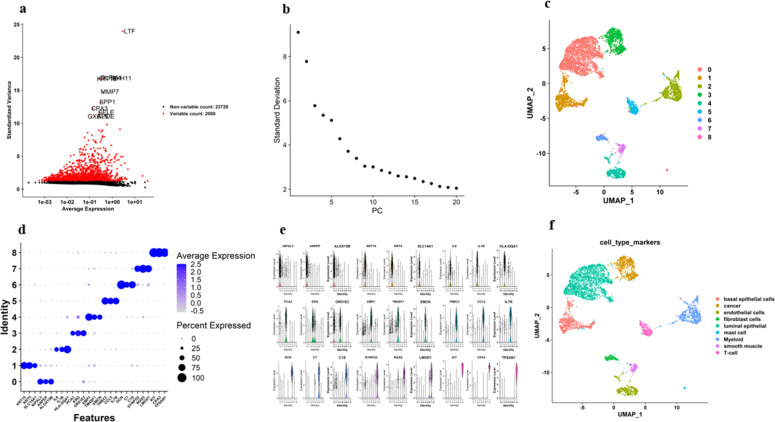
**Clustering and annotation of scRNA-seq data.** (a) Identification of highly variable genes across cells, with variable genes highlighted in red. (b) Standard deviation of PCs, showing the variation captured by each PC.(c) Unsupervised clustering of cells visualized using UMAP, with cells grouped into nine clusters.(d) Dot plot displaying marker gene expression across clusters, with dot size demonstrating the percentage of cells expressing each gene and color representing average expression.(e) Feature plots displaying the distribution of marker gene expression across clusters.(f) UMAP visualization of annotated cell types based on marker gene expression.

We utilized the Seurat package [47] for the unsupervised clustering of single-cell gene expression data (see Section 2). A total of 20,488 cells were grouped into nine distinct clusters, as shown in Fig. 8c. Cell distribution across clusters was as follows: Cluster 0 – 2059 cells; Cluster 1 – 736 cells; Cluster 2 – 686 cells; Cluster 3 – 671 cells; Cluster 4 – 413 cells; Cluster 5 – 293 cells; Cluster 6 – 160 cells; Cluster 7 – 157 cells; and Cluster 8 – 36 cells. Clusters were annotated based on literature and marker gene expression (Fig. 8d and Table 3). Cluster 0 (luminal epithelial cells) expressed ANPEP, NIPAL3, and ALOX15B; Cluster 1 (basal epithelial cells) expressed KRT15, KRT5, and SLC14A1; Cluster 2 (myeloid cells) expressed IL8, IL1B, and HLA-DQA1; and Cluster 3 (cancer cells) expressed PCA3, ERG, and OR51E2. Cluster 4 (endothelial cells) expressed EMP1, TM4SF1, and EMCN; Cluster 5 (T-cells) expressed TRBC2, CCL5, and IL7R; Cluster 6 (fibroblasts) expressed DCN, C7, and C1S; Cluster 7 (smooth muscle cells) expressed SYNPO2, RGS5, and LMOD1; and Cluster 8 (mast cells) expressed KIT, CPA3, and TPSAB1.

**Table 3:** Distribution of Cell Clusters and Cell Markers.

Cell Cluster	Cell Marker	Cell Type
0	NIPAL3, ANPEP, ALOX15B, NEFH, GCNT2, VEGFA	Luminal epithelial
1	KRT15, KRT5, SLC14A1, LAMB3, NTN4, KRT19	Basal epithelial cells
2	IL8, IL1B, HLA-DQA1, IFI30, LYZ, CYBB	Myeloid
3	PCA3, ERG, OR51E2, MYO6, STEAP4, SLC25A6	Cancer
4	EMP1, TM4SF1, EMCN, VWF, SELE, IFI27	Endothelial cells
5	TRBC2, CCL5, IL7R, CD8A, ETS1, SPOCK2	T-cell
6	DCN, C7, C1S, FBLN1, COL1A2, IGF1	Fibroblast cells
7	SYNPO2, RGS5, LMOD1, MYH11, ACTA2, TAGLN	Smooth muscle
8	KIT, CPA3, TPSAB1, IL1RL1, HPGDS, LMNA	Mast cell

Feature plots (Fig. 8) and UMAP visualizations (Fig. 8f) illustrate marker gene expression and annotated cell types in low-dimensional space. Pairwise comparisons between cell clusters 3 and 4 identified 590 significant DEGs (adjusted *p*-value 〈 0.05, |logFC| 〉 1). Tumor cells showed upregulation of PCA3, ERG, and other oncogenic markers, while basal epithelial cells were enriched for KRT5 and KRT15. PCA3 mRNA was overexpressed 66-fold in PCa compared to benign tissue [60]. ERG protein overexpression indicates ERG gene rearrangement in PCa [61], and PSGR (OR51E2) is overexpressed in PCa [62]. Key immune-related genes, such as IL1B in myeloid cells and CCL5 in T cells, were differentially expressed, reflecting the immune landscape of prostate cancer. CCL5 promotes invasion by increasing MMP-2/9 secretion and activating ERK and Rac signaling [63]. IL-1β enhances myeloid-derived suppressor cell (MDSC) accumulation, suppressing cytotoxic T cells and creating an immune-suppressive microenvironment [64].

**Fig. 9:**
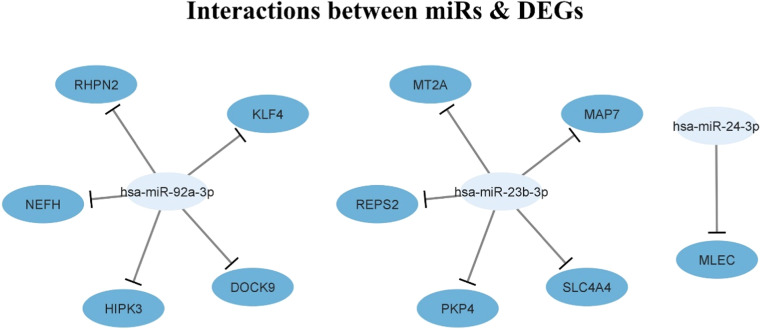
Visualizing the interactions between miRNAs and DEGs using Cytoscape.

Note: This representation focuses solely on the interaction between the selected miRNA panel and DEGs for clarity.

MiRNA-target predictions for DEGs revealed key miRNAs, including miR-92a-3p, miR-23b-3p, and miR-24–3p, as regulators of oncogenic and tumor suppressor genes. Cytoscape visualization showed a dense interaction network (Fig. 9), with miR-92a-3p regulating SOX4 [65], miR-24–3p enhancing apoptosis by targeting p27, p16, and FSCN1 [66], and miR-23b inhibiting Src kinase and Akt [67], highlighting miRNA-mediated pathways as critical to PCa biology.

### Experimental results

#### Expression analysis of candidate miRNAs

**Fig. 10:**
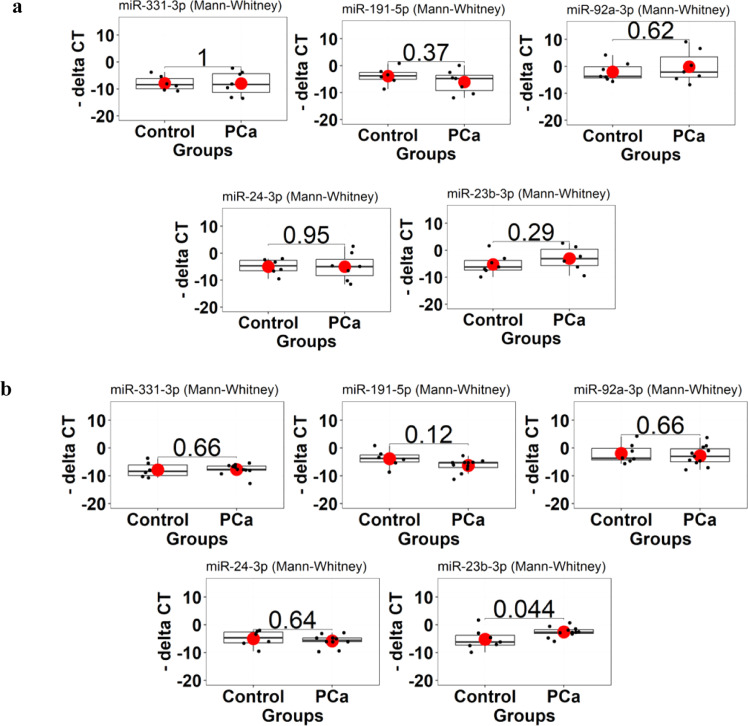
**Differentially expressed Urinary miRNAs in BPH versus PCa patients.** a) There were no significant differences in the expression levels of miR-331–3p, miR-191–5p, miR-92a-3p, miR-23b-3p, and miR-24–3p between BPH and low-grade PCa urine samples (*P* > 0.05). b) In contrast, miR-23b-3p exhibited significant deregulation in high-grade PCa compared to BPH (*P* = 0.044), suggesting its potential utility as a biomarker for high-grade PCa.

Comparison of miR-331–3p, miR-191–5p, miR-92a-3p, miR-23b-3p, and miR-24–3p expression levels between BPH (*n* = 7) and low-grade urine samples (*n* = 6) showed no significant differences (*P* > 0.05; Fig. 10a). However, in the high-grade group (*n* = 12) compared to BPH (*n* = 7), miR-23b-3p showed a significant difference, while miR-331–3p, miR-191–5p, miR-92a-3p, and miR-24–3p did not (Fig. 10b).

#### Evaluation of biomarker performance for PCa

**Fig. 11:**
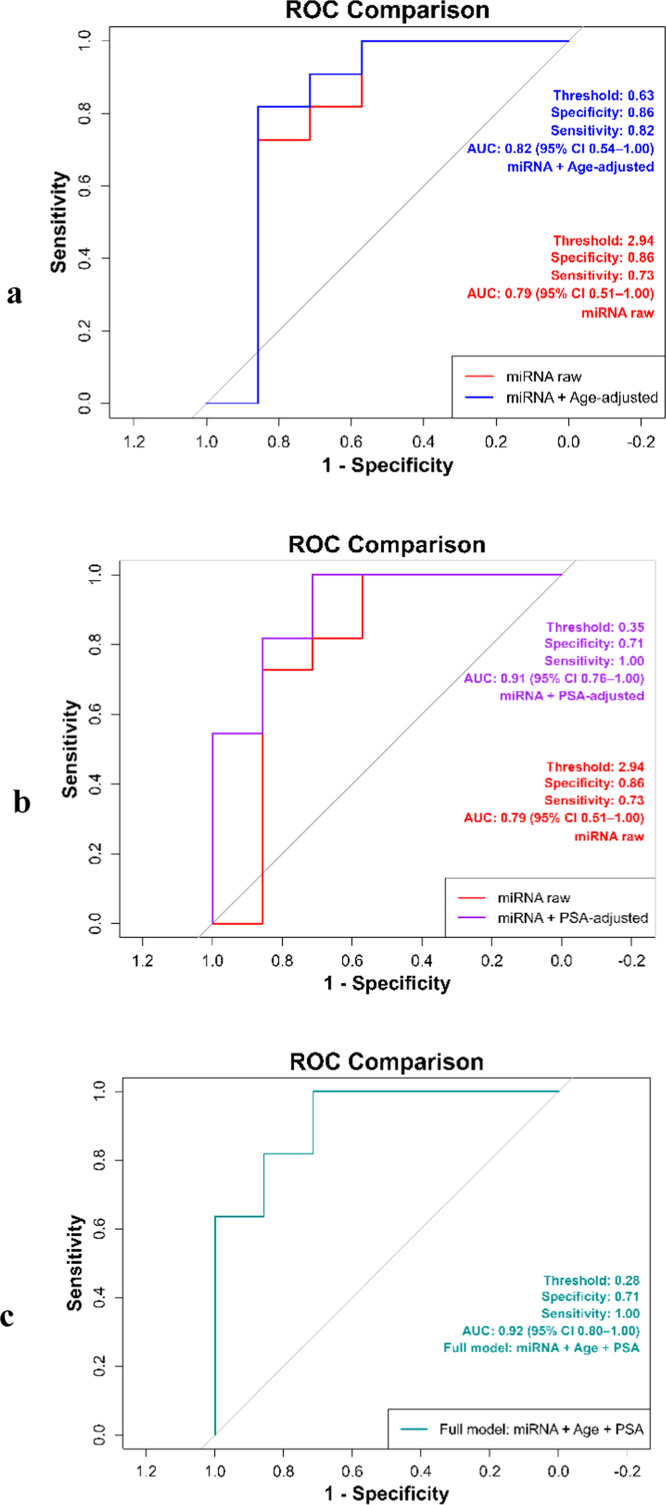
**ROC curve analysis of urinary miR-23b-3p expression levels in PCa patients.** (a) The red line represents the raw delta Ct values, while the blue line shows the age-adjusted model. (b) (c) Combined model incorporating miR-23b-3p ΔCt, age, and PSA. AUC values are 0.79 (95% CI: 0.51–1.00). for the raw model, 0.82 (95% CI: 0.54–1.00), for the age-adjusted model, 0.91 (95% CI: 0.76–1.00) for the PSA-adjusted model, and 0.92 (95% CI: 0.80–1.00) for the combined model.

To evaluate the diagnostic potential of miR-23b-3p for PCa, we performed ROC curve analysis, which assesses the assay's ability to distinguish between patients with BPH and those with PCa. This method provides a measure of sensitivity (true positive rate) and specificity (true negative rate), reported as numeric values without NA to ensure clarity. Additionally, the area under the curve (AUC) is reported alongside 95% confidence intervals (CI) to indicate the precision and statistical significance of the diagnostic performance. Fig. 11a illustrates the ROC curves for both the raw deltaCt values (red line) and the age-adjusted logistic regression model (blue line). The analysis revealed that miR-23b-3p expression demonstrated good discriminatory power, with an area under the curve (AUC) of 0.79 (95% CI: 0.51–1.00). The specificity and sensitivity values were 0.86 (95% CI: 0.42–1.00) and 0.73 (95% CI: 0.39–0.94), respectively, indicating a reliable ability to differentiate PCa from BPH (*p* < 0.05). When age is included as a covariate in a logistic regression model, the diagnostic performance of miR-23b-3p improves. The model achieves an AUC of 0.82(95% CI: 0.54–1.00), a sensitivity of 0.82(95% CI: 0.48–0.98), and a specificity that remains unchanged at 0.86(95% CI: 0.42–1.00). This indicates a modest additive effect of age in differentiating PCa from BPH. These results suggest that miR-23b-3p may serve as a potential urinary biomarker for the detection of PCa. ROC analysis for PSA alone was conducted to evaluate its standalone diagnostic potential, resulting in an AUC of 0.86(95% CI: 0.66–1.00), with a sensitivity of 0.82 (95% CI: 0.48–0.98) and a specificity of 0.86 (95% CI: 0.42–1.00). Including PSA as a covariate in a logistic regression model enhances the diagnostic performance of miR-23b-3p The PSA-adjusted model achieves an AUC of 0.91(95% CI: 0.76–1.00), showing a sensitivity of 100%(95% CI: 0.72–1.00) and a specificity of 71.4% (95% CI: 0.29–0.96). This indicates that PSA significantly improves the ability of the biomarker to differentiate PCa from BPH. Fig. 11b displays the ROC curves for the raw miR-23b-3p values (red line) and the PSA-adjusted model (purple line). The final evaluation of the combined multivariable model included miR-23b-3p ΔCt, age, and PSA levels (as shown in Fig. 11c). The predicted probabilities from this model were used to create a composite ROC curve, which resulted in an AUC of 0.92 (95% confidence interval: 0.80–1.00). This model demonstrated a sensitivity of 1.00 (95% CI: 0.72–1.00) and a specificity of 0.71 (95% CI: 0.29–0.96). These findings indicate that integrating all three variables significantly enhances the ability to differentiate PCa from BPH.

## Discussion

In this study, miR-23b-3p showed promising potential as a urinary biomarker for distinguishing PCa from BPH, building upon its prior identification through integrative multi-omics bioinformatics analyses and subsequent experimental validation, and supporting the concept that integrative biomarker discovery strategies can improve diagnostic discrimination beyond single-marker approaches. It demonstrated diagnostic performance with an AUC of 0.79 (95% CI: 0.51–1.00), specificity of 0.86 (95% CI: 0.42–1.00), and sensitivity of 0.73 (95% CI: 0.39–0.94) Although the confidence intervals are relatively wide, reflecting the limited cohort size, the lower bound of the AUC confidence interval remains above 0.5, supporting a discriminatory performance beyond random classification. Incorporating age as a covariate in a logistic regression model resulted in a modest improvement in diagnostic performance, raising the AUC by 0.82 (95% CI: 0.54–1.00) while maintaining specificity at 0.86 (95% CI: 0.42–1.00). This limited increase suggests that age acts as a weak but biologically plausible confounder rather than a primary driver of classification performance. When evaluated independently, PSA showed reasonable performance, Importantly, inclusion of PSA as a covariate substantially enhanced the discriminatory ability of miR-23b-3p, increasing the AUC of miR-23b-3p to 0.91(95% CI: 0.76–1.00), with a sensitivity of 1.00(95% CI: 0.72–1.00) and a specificity of 0.71(95% CI: 0.29–0.96).This synergistic effect likely reflects the complementary diagnostic properties of PSA and urinary miR-23b-3p, with PSA contributing sensitivity and miR-23b-3p improving specificity. The highest diagnostic performance was observed in the combined multivariable model incorporating miR-23b-3p ΔCt, age, and PSA, which achieved an AUC of 0.92 (95% CI: 0.80–1.00), also achieving a sensitivity of 1.00 (95% CI: 0.72–1.00) and a specificity of 0.71(95% CI: 0.29–0.96).While this model demonstrates strong discriminatory potential, these findings should be interpreted as exploratory given the sample size and require validation in larger, independent cohorts. This suggests that both PSA and age enhance biomarker performance. These preliminary results highlight the need for larger studies and PSA- or age-adjusted models to validate miR-23b-3p The diagnostic performance without prior DRE suggests it could be feasible for routine clinical use and is patient-friendly.

This study has several limitations worth noting. The relatively small sample size and strict inclusion criteria may limit the generalizability of the findings. While catheterized urine collection was used to ensure sample integrity, it may not fully represent standard non-invasive clinical practices. The PCa group included only patients with confirmed histopathological diagnoses, whereas the BPH group included patients with negative pathology results after OP surgery or TURP. Given the 20–25% false-negative rate of prostate biopsies, some PCa patients could have undetected malignancies. Although excluding such cases enhanced internal validity, it also reduced the final cohort size and prolonged the sample collection period. Taken together, these considerations highlight that, while the current findings are internally valid, external validation in larger, independent cohorts is necessary to confirm and generalize these results. Several methodological considerations should be noted regarding the machine learning analyses. Candidate miRNAs were pre-selected based on differential expression, cross-dataset consistency, and biological relevance, which reduced the risk of data-driven feature selection bias. However, performance estimates were derived from repeated random 60/40 train–test splits, which may not fully account for overfitting in the context of limited sample sizes. Accordingly, the reported classification metrics should be interpreted as preliminary and hypothesis-generating. The development of a clinically robust diagnostic model will require more stringent validation approaches, including k-fold cross-validation and evaluation in larger, independent cohorts. Within these constraints, our findings suggest that urinary miR-23b-3p represents a promising non-invasive biomarker candidate for prostate cancer, meriting further investigation to clarify its diagnostic value.

Current prostate cancer (PCa) screening strategies, including PSA testing, DRE, MRI, and systematic biopsy, have significant limitations. PSA testing has a high false-positive rate (20–50%) and can also be elevated in benign conditions, while aggressive PCa may show low PSA levels [68,69]. DRE is subjective and invasive, with only 2% of suspicious findings leading to significant diagnoses [70]. MRI improves detection but suffers from low positive predictive values and inter-reader variability, highlighting the need for better patient selection and AI integration [71]. Systematic biopsy, the current gold standard, poses risks such as infection and offers only a limited view of tumor heterogeneity [72,73]. These challenges stress the need for complementary non-invasive molecular biomarkers.

Liquid biopsy provides a minimally invasive method for real-time monitoring of molecular changes associated with tumors [74]. In addition to circulating neoplastic cells and DNA fragments found in bodily fluids, exosomes from liquid biopsies provide valuable insights into tumor molecular composition. Urinary liquid biopsy is a promising method for detecting prostate cancer. Potential serum biomarkers that could enhance precision medicine include androgen receptor variants, bone metabolism markers, neuroendocrine indicators, and metabolite biomarkers. Analyzing exosomes from blood, urine, and semen in prostate cancer patients offers significant advantages for identifying potential biomarkers [75]. Compared to protein-based biomarkers, RNA-based approaches, such as those focusing on microRNAs and long non-coding RNAs, offer advantages in terms of sensitivity, specificity, and cost-effectiveness. The dynamic nature of RNA supplies valuable insights into cellular regulation and states, suggesting a more nuanced understanding of tumor biology than DNA alone [11].

Urinary miRNAs, in particular, are attractive due to their stability, non-invasive collection, and direct contact with prostate tissue. However, challenges such as lack of standardized protocols, sample contamination, and PCa heterogeneity hinder their clinical translation [58,[76], [77], [78]]. This study analyzed five urinary miRNAs (miR-331–3p, miR-191–5p, miR-92a-3p, miR-24–3p, and miR-23b-3p) in PCa patients without prior DRE, revealing significant downregulation of **miR-23b-3p** in PCa compared to BPH. This is particularly relevant given the limitations of PSA in differentiating between these conditions.

This aligns with previous studies showing miR-23b’s role in suppressing metastasis and its downregulation in castration-resistant prostate cancer (CRPC) [[79], [80], [81]]. While prior research focused on miR-23b’s prognostic and metastatic roles, our study highlights its diagnostic potential, offering a non-invasive alternative for early PCa detection. These findings emphasize the need for larger, multicenter studies to validate the clinical utility of miR-23b-3p and explore its integration into diagnostic workflows.

## Conclusion

This study highlights miR-23b-3p as a promising non-invasive biomarker for PCa due to its stability in biofluids and tumor-reflective properties. Despite challenges in standardization, urine-based PCa-specific miRNAs show potential for biomarker discovery. Further research is needed to standardize methods and integrate these biomarkers into clinical practice for early detection, prognosis, and personalized treatment.

## Ethics approval and consent to participate

Human Ethics and Consent to Participate declarations: Not applicable, as written consent was formally waived by the ethics committees. Nevertheless, verbal informed consent was obtained from all participants in accordance with the ethical standards outlined in the Declaration of Helsinki (1964) and its subsequent amendments.

## Clinical trial number

Not applicable. This study is an observational study and not a clinical trial.

The study protocol was approved by the Ethics Committees of the Urology and Nephrology Research Center (IR.SBMU.UNRC.REC.1401.027, 25/12/2022) and Tarbiat Modares University (IR.MODARES.REC.1401.041). The study posed minimal risk, involving only the collection of biological samples and routine clinical data, which was fully anonymized. Participants were informed about the study's objectives, procedures, and potential risks in their native language, and were assured of data anonymity. All provided verbal consent for participation and publication of anonymized data. Participation was entirely voluntary.

## Consent for publication

Not applicable.

## Funding

This research was funded by The 10.13039/100006280Urology & Nephrology Research Center Foundation (project number: 43003406) and Tarbiat Modarres University.

## Availability of data and materials

The datasets generated and analyzed during this study are available from the corresponding author (sjmowla@modares.ac.ir) on reasonable request. The microarray data for PCa patients and controls are publicly available in the GEO database, accessible using the following accession numbers: GSE36802, GSE23022, GSE45604, GSE112264, GSE159177, GSE138740, and GSE86474. Additionally, the RNA-seq and single-cell datasets can also be found in GEO, with accession numbers GSE166697 and GSE176031, respectively. Furthermore, the miRNA-seq dataset is publicly accessible through the TCGA program under the accession number TCGA-PRAD.

## Declaration of generative AI and AI-Assisted technologies in the writing process

While preparing this work, the authors used DeepSeek Chat (an AI language model developed by DeepSeek) and Grammarly for writing assistance, paraphrasing, and grammar checking. All AI-generated or AI-edited content was carefully reviewed, revised, and approved by the authors, who assume full responsibility for the publication’s accuracy and originality.

## CRediT authorship contribution statement

**Leila Asadi Samani:** Investigation, Data curation, Formal analysis, Methodology, Validation, Visualization, Writing – original draft, Writing – review & editing, Conceptualization. **Saeid Rahmani:** Software, Formal analysis, Validation, Methodology, Visualization, Investigation, Data curation. **Amir Hossein Kashi:** Methodology, Validation, Visualization, Formal analysis, Investigation, Data curation. **Seyed Amir Mohsen Ziaee:** Supervision, Project administration, Resources, Investigation, Data curation, Formal analysis, Methodology, Validation, Visualization. **Seyed Javad Mowla:** Supervision, Project administration, Resources, Validation, Investigation, Data curation, Formal analysis, Methodology, Visualization, Funding acquisition, Writing – review & editing.

## Declaration of competing interest

The authors declare that they have no known competing financial interests or personal relationships that could have appeared to influence the work reported in this paper.

## Appendix Supplementary materials

Image, application 1
