# Supplementary material for: Integrative analysis of urinary microRNAs for prostate cancer detection: A proof-of-concept study
Source: Transl Oncol. 2026 Apr 7;67:102745. doi: 10.1016/j.tranon.2026.102745 (PMC13090718; doi:10.1016/j.tranon.2026.102745)

**Table S1. The sequences of the stem-loop primers utilized in this study.**

| Genes/miRs | Stem-loop primer | Forward Primer | Reverse Primer |
| --- | --- | --- | --- |
| miR-331-3p | GAAAGAAGGCGAGGAGCAGATCGAGGAAGAAGACGGAAG  AATGTGCGTCTCGCCTTCTTTCTTCTAG | CATGATGCCCCTGGGCCTA | CGAGGAAGAAGACGGAAGAAT |
| miR-191-5p | GAAAGAAGGCGAGGAGCAGATCGAGGAAGAAGACGGAAG  AATGTGCGTCTCGCCTTCTTTC CAGCTG | CACGCCAACGGAATCCCA | CGAGGAAGAAGACGGAAGAAT |
| miR-92a-3p | GAAAGAAGGCGAGGAGCAGATCGAGGAAGAAGACGGAAG  AATGTGCGTCTCGCCTTCTTTC ACAGGC | GAGCGTATTGCACTTGTCCC | CGAGGAAGAAGACGGAAGAAT |
| miR-24-3p | GTCGTATCCAGTGCAGGGTCCGAGGTATTCGCACTGGATACGAC CTGTTC | CCGATGGCTCAGTTCAGCA | CCAGTGCAGGGTCCGAGGTA |
| miR-23b-3p | GTCGTATCCAGTGCAGGGTCCGAGGTATTCGCACTGGATACGAC  GTGGTA | CCACCGATCACATTGCCAGG | CCAGTGCAGGGTCCGAGGTA |
| 5S rRNA | - | GTCTACGGCCATACCACCCTG | AAAGCCTACAGCACCCGGTAT |
| M13 | - | GTAAACGACGGCCAGT | CAGGAAACAGCTATGAG |

a: GSE36802


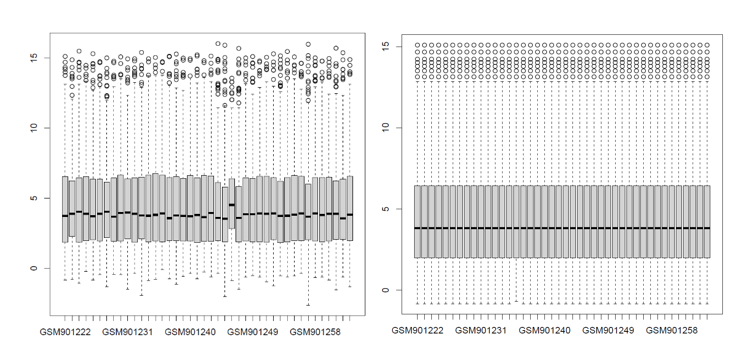


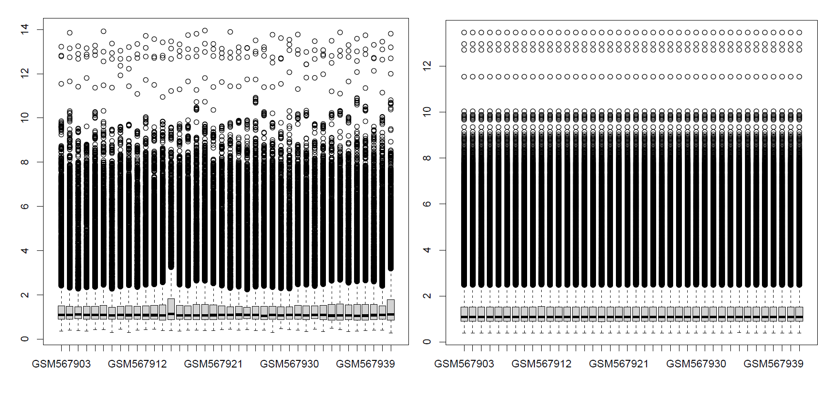


b: GSE23022

c: GSE45604


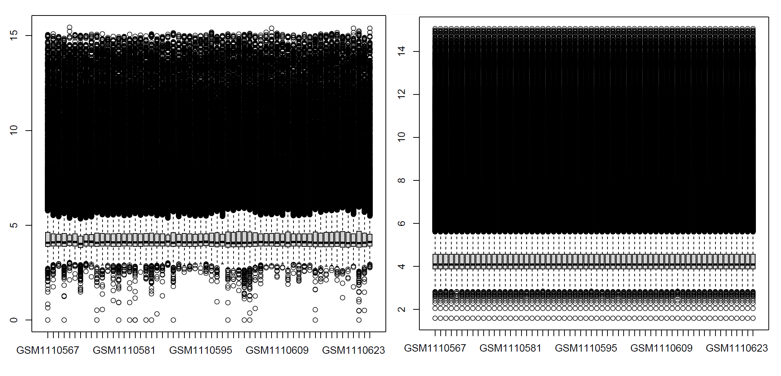


d: GSE112264


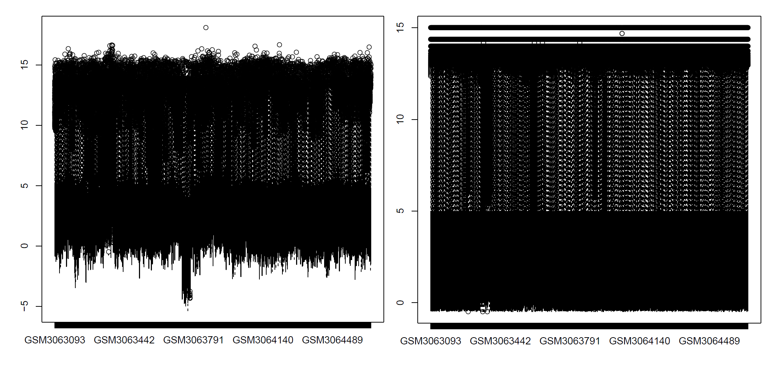


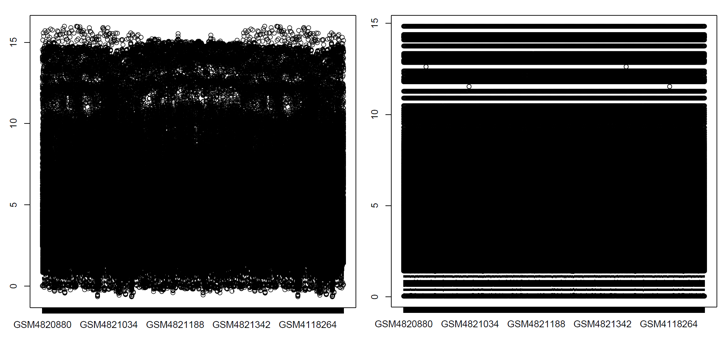


e: GSE159177, GSE138740

f: GSE86474


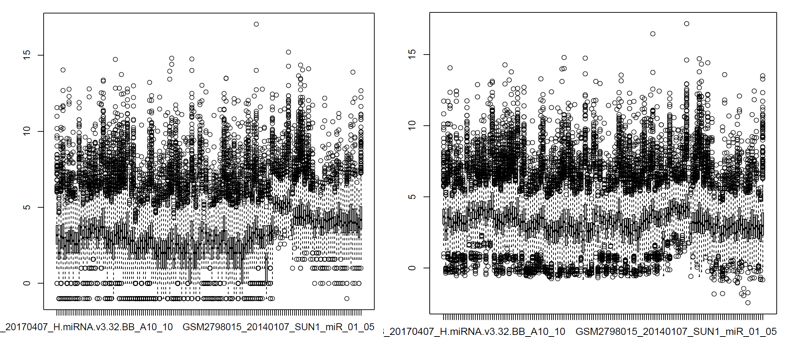


**Figure S1: Boxplot of the relative log expression (RLE) values for PCa sample arrays, shown before (left) and after (right) batch correction.** The RLE distribution is centered around zero, indicating a significant reduction in unwanted variation. Note: For GSE86474, Figure 1f shows the boxplot before (left) and after (right) correcting for the batch effect

Table S2: List of Differentially Expressed miRNAs in Prostate Cancer from GSE36802.

| miRNA ID | logFC | adj.P.Val |
| --- | --- | --- |
| hsa-mir-153-1 | 2.068097798 | 0.000174328 |
| hsa-mir-375 | 1.2383805 | 4.31E-05 |
| hsa-mir-1244 | 1.187106742 | 0.019923404 |
| hsa-mir-148a | 1.062888162 | 4.65E-05 |
| hsa-mir-222 | -1.201593195 | 9.15E-05 |
| hsa-mir-224 | -1.234738606 | 0.004714745 |
| hsa-mir-204 | -1.267990985 | 0.001437188 |
| hsa-mir-145 | -1.284326233 | 1.42E-05 |
| hsa-mir-133b | -1.433487535 | 0.000179902 |
| hsa-mir-376c | -1.492483455 | 0.000117636 |
| hsa-mir-221 | -1.553934484 | 1.46E-06 |
| hsa-mir-133a-1 | -1.663538211 | 9.15E-05 |
| hsa-mir-455 | -1.665902963 | 8.53E-07 |
| hsa-mir-31 | -1.667662952 | 0.000109114 |
| hsa-mir-205 | -3.327448767 | 0.000109114 |

Table S3: List of Differentially Expressed miRNAs in Prostate Cancer from GSE23022

| miRNA ID | logFC | adj.P.Val |
| --- | --- | --- |
| hsa-mir-375 | 1.951761939 | 0.003601537 |

**Table S4: List of Differentially Expressed miRNAs in Prostate Cancer from GSE45604.**

| miRNA ID | logFC | adj.P.Val |
| --- | --- | --- |
| hsa-miR-768-3p | 1.345744851 | 0.023862289 |
| hsa-mir-375 | 1.33488026 | 0.001861767 |
| hsa-mir-1975 | 1.121872717 | 0.036180553 |
| hsa-mir-183 | 1.083879733 | 0.011665177 |
| hsa-mir-720 | 1.082948535 | 0.048839488 |
| hsa-mir-182 | 1.0764811 | 0.004002183 |
| hsa-mir-449a | 1.065666096 | 0.000238006 |
| hsa-miR-30c-2-star | -1.047033663 | 0.041633471 |
| hsa-mir-187 | -1.083425318 | 0.029368816 |
| hsa-miR-1 | -1.393880754 | 0.020918851 |
| hsa-miR-133a | -1.403712213 | 0.00256147 |

**Table S5: List of Differentially Expressed miRNAs in prostate cancer from GSE112264 compared to non-cancer controls.**

| miRNA ID | logFC | adj.P.Val |
| --- | --- | --- |
| hsa-miR-1246 | 6.668561211 | 1.89E-79 |
| hsa-miR-191-5p | 5.289155014 | 5.45E-84 |
| hsa-miR-6131 | 5.017616554 | 4.34E-69 |
| hsa-miR-451a | 4.210084904 | 1.32E-43 |
| hsa-miR-29b-3p | 3.924613409 | 1.88E-38 |
| hsa-miR-4480 | 3.910835596 | 1.44E-40 |
| hsa-miR-4771 | 3.833465711 | 3.42E-36 |
| hsa-miR-124-3p | 3.720846727 | 6.95E-38 |
| hsa-miR-320e | 3.675423395 | 3.49E-32 |
| hsa-miR-4755-3p | 3.618191664 | 2.49E-20 |
| hsa-miR-1290 | 3.547480358 | 3.31E-50 |
| hsa-miR-6073 | 3.491300774 | 1.79E-18 |
| hsa-miR-17-3p | 3.279620079 | 8.70E-49 |
| hsa-miR-3194-3p | 3.16008487 | 6.10E-29 |
| hsa-miR-29b-1-5p | 3.124795715 | 4.25E-14 |
| hsa-miR-4718 | 3.077205574 | 3.36E-38 |
| hsa-miR-23b-3p | 3.024910181 | 4.65E-16 |
| hsa-miR-4757-5p | 3.022828782 | 4.69E-20 |
| hsa-miR-5100 | 3.00853536 | 1.71E-82 |
| hsa-miR-4666a-5p | 2.992617989 | 7.62E-15 |
| hsa-miR-103a-3p | 2.927763732 | 4.04E-12 |
| hsa-miR-4663 | 2.916033578 | 3.23E-15 |
| hsa-miR-3688-5p | 2.909269369 | 1.09E-15 |
| hsa-miR-22-3p | 2.89676372 | 4.18E-16 |
| hsa-miR-2467-3p | 2.836122792 | 1.70E-40 |
| hsa-miR-4658 | 2.825880667 | 1.88E-16 |
| hsa-miR-4529-5p | 2.822345424 | 1.15E-15 |
| hsa-miR-4525 | 2.817174845 | 1.44E-40 |
| hsa-miR-4789-5p | 2.804827084 | 3.11E-12 |
| hsa-miR-614 | 2.742882292 | 3.13E-84 |
| hsa-miR-6501-3p | 2.653645122 | 3.00E-16 |
| hsa-miR-320d | 2.625903023 | 1.49E-15 |
| hsa-miR-4708-3p | 2.621278313 | 4.08E-35 |
| hsa-miR-4423-5p | 2.525453101 | 2.81E-11 |
| hsa-miR-107 | 2.522779158 | 2.80E-09 |
| hsa-miR-6736-5p | 2.501112483 | 4.21E-20 |
| hsa-miR-4652-5p | 2.491632585 | 3.13E-11 |
| hsa-miR-6717-5p | 2.490972679 | 4.64E-44 |
| hsa-miR-4434 | 2.485631589 | 4.86E-09 |
| hsa-miR-4515 | 2.469782324 | 3.86E-32 |
| hsa-miR-519d-5p | 2.466523158 | 5.14E-15 |
| hsa-miR-4633-3p | 2.456693711 | 5.13E-09 |
| hsa-miR-1307-3p | 2.418864853 | 3.27E-29 |
| hsa-miR-3192-5p | 2.401932857 | 2.97E-11 |
| hsa-miR-23a-3p | 2.38607117 | 1.95E-08 |
| hsa-miR-4740-5p | 2.369501484 | 4.89E-20 |
| hsa-miR-4700-5p | 2.355156847 | 2.50E-12 |
| hsa-miR-151b | 2.354929114 | 2.44E-09 |
| hsa-miR-99a-5p | 2.343184985 | 5.30E-09 |
| hsa-miR-4777-3p | 2.32505093 | 6.15E-10 |
| hsa-miR-4454 | 2.319045164 | 7.10E-85 |
| hsa-miR-4490 | 2.316357061 | 6.98E-09 |
| hsa-miR-26a-5p | 2.314509873 | 1.90E-09 |
| hsa-miR-24-1-5p | 2.274896196 | 6.10E-09 |
| hsa-miR-3160-5p | 2.261463827 | 1.16E-15 |
| hsa-miR-4727-3p | 2.259063864 | 3.35E-28 |
| hsa-miR-6839-5p | 2.258967471 | 1.10E-14 |
| hsa-miR-4448 | 2.240026784 | 2.79E-22 |
| hsa-miR-130a-3p | 2.238992665 | 2.55E-09 |
| hsa-miR-5586-3p | 2.217974452 | 6.65E-10 |
| hsa-miR-4299 | 2.208751545 | 1.10E-09 |
| hsa-miR-4635 | 2.202751358 | 2.10E-13 |
| hsa-miR-3064-5p | 2.184773054 | 1.65E-09 |
| hsa-miR-151a-5p | 2.15000592 | 2.44E-09 |
| hsa-miR-5692b | 2.142371627 | 5.32E-08 |
| hsa-miR-4439 | 2.128984425 | 7.31E-09 |
| hsa-miR-4532 | 2.122473551 | 4.64E-34 |
| hsa-miR-24-3p | 2.117073946 | 5.68E-19 |
| hsa-miR-519e-5p | 2.117036773 | 2.29E-13 |
| hsa-miR-520f-5p | 2.116043391 | 5.85E-10 |
| hsa-miR-140-3p | 2.114668094 | 4.01E-08 |
| hsa-miR-654-5p | 2.10534503 | 2.34E-14 |
| hsa-miR-342-5p | 2.098701469 | 2.00E-27 |
| hsa-miR-4633-5p | 2.080645071 | 2.79E-08 |
| hsa-miR-4650-3p | 2.071717409 | 4.10E-08 |
| hsa-miR-518d-3p | 2.063507965 | 1.01E-07 |
| hsa-miR-548b-3p | 2.062789555 | 3.18E-07 |
| hsa-miR-4783-3p | 2.048812282 | 4.39E-22 |
| hsa-miR-586 | 2.04871388 | 5.02E-07 |
| hsa-miR-3156-5p | 2.048238897 | 3.98E-18 |
| hsa-miR-6760-5p | 2.046867867 | 4.01E-18 |
| hsa-miR-4732-5p | 2.028101361 | 3.92E-13 |
| hsa-miR-3617-5p | 2.011828433 | 2.82E-08 |
| hsa-miR-320b | 1.974626163 | 2.40E-22 |
| hsa-miR-8059 | 1.9629803 | 4.05E-49 |
| hsa-miR-4295 | 1.930763929 | 1.88E-06 |
| hsa-miR-320c | 1.918255279 | 1.65E-10 |
| hsa-miR-524-5p | 1.91002648 | 2.98E-07 |
| hsa-miR-8073 | 1.903553438 | 1.51E-81 |
| hsa-miR-4795-3p | 1.894766916 | 6.05E-06 |
| hsa-miR-4710 | 1.893185502 | 5.03E-18 |
| hsa-miR-629-5p | 1.889020871 | 2.63E-06 |
| hsa-miR-2114-3p | 1.887421739 | 1.12E-07 |
| hsa-miR-184 | 1.880723262 | 1.38E-07 |
| hsa-miR-5701 | 1.862844644 | 3.17E-07 |
| hsa-miR-6863 | 1.861459022 | 1.79E-06 |
| hsa-miR-551b-5p | 1.858403227 | 1.80E-15 |
| hsa-miR-5706 | 1.857517539 | 1.24E-07 |
| hsa-miR-3155a | 1.82021087 | 1.45E-07 |
| hsa-miR-28-5p | 1.815655289 | 1.36E-06 |
| hsa-miR-3121-5p | 1.802341807 | 3.25E-06 |
| hsa-miR-4489 | 1.79984352 | 1.97E-12 |
| hsa-miR-1233-5p | 1.794681055 | 9.99E-41 |
| hsa-miR-4724-3p | 1.765100644 | 2.77E-06 |
| hsa-miR-4259 | 1.762533521 | 9.41E-15 |
| hsa-miR-379-5p | 1.752747215 | 9.19E-07 |
| hsa-miR-8060 | 1.75052241 | 1.63E-07 |
| hsa-miR-4774-5p | 1.732012735 | 1.16E-06 |
| hsa-miR-4475 | 1.722868251 | 2.39E-06 |
| hsa-miR-4300 | 1.713115409 | 1.73E-06 |
| hsa-miR-15b-5p | 1.7123263 | 1.23E-05 |
| hsa-miR-668-5p | 1.692312457 | 2.68E-11 |
| hsa-miR-6864-3p | 1.688696699 | 3.78E-05 |
| hsa-miR-4444 | 1.679050803 | 1.08E-05 |
| hsa-miR-495-5p | 1.671794052 | 7.71E-06 |
| hsa-miR-1343-3p | 1.653462864 | 5.42E-51 |
| hsa-miR-4514 | 1.640132133 | 7.73E-06 |
| hsa-miR-4776-5p | 1.639318631 | 4.52E-08 |
| hsa-miR-4538 | 1.631852533 | 3.46E-06 |
| hsa-miR-4694-5p | 1.62388065 | 3.75E-05 |
| hsa-miR-4744 | 1.622005993 | 4.29E-05 |
| hsa-miR-3977 | 1.610057005 | 1.26E-05 |
| hsa-miR-27b-3p | 1.608436998 | 7.08E-05 |
| hsa-miR-4770 | 1.603394262 | 2.81E-06 |
| hsa-miR-26a-2-3p | 1.598437127 | 6.84E-05 |
| hsa-miR-92a-3p | 1.595535399 | 7.65E-11 |
| hsa-miR-7975 | 1.587751919 | 2.13E-40 |
| hsa-miR-423-3p | 1.584298981 | 4.78E-06 |
| hsa-miR-4436b-3p | 1.583638958 | 1.61E-05 |
| hsa-miR-548ar-5p | 1.579447333 | 2.34E-05 |
| hsa-miR-520h | 1.565763731 | 0.00010475 |
| hsa-miR-1260b | 1.562056149 | 2.73E-65 |
| hsa-miR-6838-3p | 1.555580429 | 7.30E-06 |
| hsa-miR-5692a | 1.555204121 | 1.14E-05 |
| hsa-miR-5704 | 1.550943413 | 1.11E-05 |
| hsa-miR-4531 | 1.549575296 | 2.68E-05 |
| hsa-miR-650 | 1.548290957 | 2.10E-07 |
| hsa-miR-5092 | 1.536025257 | 4.47E-05 |
| hsa-miR-26b-5p | 1.526475406 | 0.000103965 |
| hsa-miR-411-5p | 1.526278531 | 7.69E-05 |
| hsa-miR-4317 | 1.524581811 | 1.48E-05 |
| hsa-miR-4754 | 1.523877659 | 1.27E-05 |
| hsa-miR-320a | 1.520796892 | 1.02E-28 |
| hsa-miR-588 | 1.51745721 | 2.53E-05 |
| hsa-miR-6747-3p | -1.500327869 | 2.08E-05 |
| hsa-miR-1226-3p | -1.501027563 | 2.62E-05 |
| hsa-miR-3130-5p | -1.50729059 | 1.73E-05 |
| hsa-miR-6745 | -1.509423665 | 2.83E-08 |
| hsa-miR-6499-5p | -1.513957791 | 3.23E-06 |
| hsa-miR-6761-3p | -1.514300359 | 2.46E-05 |
| hsa-miR-4714-5p | -1.519740946 | 5.59E-06 |
| hsa-miR-3190-5p | -1.526214162 | 3.79E-06 |
| hsa-miR-4526 | -1.527970225 | 3.85E-06 |
| hsa-miR-4449 | -1.530907667 | 4.57E-19 |
| hsa-miR-5088-5p | -1.531629114 | 1.39E-05 |
| hsa-miR-6852-3p | -1.533962915 | 5.40E-08 |
| hsa-miR-2278 | -1.541771514 | 3.52E-05 |
| hsa-let-7a-2-3p | -1.549086516 | 4.80E-10 |
| hsa-miR-6503-3p | -1.554721612 | 5.41E-05 |
| hsa-miR-3184-3p | -1.555990223 | 2.60E-05 |
| hsa-miR-6873-3p | -1.557996477 | 7.15E-07 |
| hsa-miR-767-3p | -1.563379775 | 2.12E-06 |
| hsa-miR-197-3p | -1.572887361 | 2.04E-06 |
| hsa-miR-4800-3p | -1.579313532 | 4.73E-06 |
| hsa-miR-6759-5p | -1.580652129 | 1.90E-05 |
| hsa-miR-3151-3p | -1.583414021 | 3.84E-06 |
| hsa-miR-6875-3p | -1.58755667 | 4.42E-06 |
| hsa-miR-6785-3p | -1.589553876 | 2.62E-07 |
| hsa-miR-4685-3p | -1.59263667 | 4.25E-06 |
| hsa-miR-6881-3p | -1.597414368 | 2.76E-07 |
| hsa-miR-6738-3p | -1.598079234 | 2.31E-07 |
| hsa-miR-3943 | -1.599327627 | 4.78E-06 |
| hsa-miR-6858-3p | -1.601722331 | 7.60E-07 |
| hsa-miR-4284 | -1.609573144 | 1.12E-05 |
| hsa-miR-3615 | -1.60972722 | 5.04E-06 |
| hsa-miR-6870-3p | -1.613918062 | 7.17E-06 |
| hsa-miR-1204 | -1.618653608 | 7.03E-09 |
| hsa-miR-512-5p | -1.619418148 | 9.53E-07 |
| hsa-miR-6774-3p | -1.622969636 | 3.83E-08 |
| hsa-miR-346 | -1.628228067 | 1.61E-06 |
| hsa-miR-6825-3p | -1.632347461 | 8.49E-09 |
| hsa-miR-4723-3p | -1.632906148 | 7.99E-07 |
| hsa-miR-6764-3p | -1.634602328 | 2.04E-09 |
| hsa-miR-4733-3p | -1.637126855 | 8.39E-06 |
| hsa-miR-3144-5p | -1.638889642 | 2.82E-06 |
| hsa-miR-485-3p | -1.65222296 | 2.10E-06 |
| hsa-miR-5002-3p | -1.652272019 | 1.28E-08 |
| hsa-miR-6734-5p | -1.656626442 | 2.86E-06 |
| hsa-miR-1231 | -1.660573963 | 3.24E-15 |
| hsa-miR-6855-5p | -1.669221143 | 2.22E-07 |
| hsa-miR-6789-3p | -1.672680143 | 3.52E-06 |
| hsa-miR-6077 | -1.676290618 | 2.22E-07 |
| hsa-miR-3689d | -1.678629499 | 9.86E-06 |
| hsa-miR-6813-3p | -1.685830722 | 1.06E-07 |
| hsa-miR-550b-3p | -1.689254215 | 1.88E-10 |
| hsa-miR-6829-3p | -1.693292028 | 4.89E-06 |
| hsa-miR-5008-3p | -1.69730079 | 4.62E-07 |
| hsa-miR-6756-3p | -1.703105396 | 3.02E-08 |
| hsa-miR-6802-3p | -1.709308207 | 4.63E-07 |
| hsa-miR-1267 | -1.710705734 | 1.18E-08 |
| hsa-miR-449b-3p | -1.716910532 | 2.17E-06 |
| hsa-miR-6720-5p | -1.731544078 | 9.95E-08 |
| hsa-miR-6824-3p | -1.73154895 | 5.58E-07 |
| hsa-miR-6892-3p | -1.733737444 | 1.13E-07 |
| hsa-miR-6825-5p | -1.73482928 | 7.48E-18 |
| hsa-miR-4701-5p | -1.738960337 | 1.91E-07 |
| hsa-miR-6891-3p | -1.740641365 | 2.00E-07 |
| hsa-miR-6877-3p | -1.742871291 | 4.80E-08 |
| hsa-miR-6760-3p | -1.750858132 | 1.33E-06 |
| hsa-miR-1273h-3p | -1.753563827 | 6.59E-08 |
| hsa-miR-6819-3p | -1.756653363 | 4.62E-07 |
| hsa-miR-6758-5p | -1.761156872 | 3.15E-07 |
| hsa-miR-636 | -1.766605965 | 6.09E-09 |
| hsa-miR-1182 | -1.774503804 | 5.20E-08 |
| hsa-miR-6754-3p | -1.788296025 | 1.09E-06 |
| hsa-let-7d-3p | -1.788613562 | 1.15E-11 |
| hsa-miR-4312 | -1.78923211 | 6.31E-07 |
| hsa-miR-572 | -1.795209052 | 2.79E-08 |
| hsa-miR-425-3p | -1.796699843 | 5.44E-07 |
| hsa-miR-4632-3p | -1.80038029 | 1.13E-07 |
| hsa-miR-6882-3p | -1.805790714 | 9.11E-07 |
| hsa-miR-5088-3p | -1.806574177 | 2.24E-07 |
| hsa-miR-194-3p | -1.80669786 | 9.50E-08 |
| hsa-miR-128-1-5p | -1.819029977 | 4.32E-17 |
| hsa-miR-877-3p | -1.823830021 | 1.36E-07 |
| hsa-miR-6813-5p | -1.82760805 | 9.98E-09 |
| hsa-miR-6784-3p | -1.82877231 | 1.11E-07 |
| hsa-miR-4251 | -1.829201489 | 1.05E-06 |
| hsa-miR-668-3p | -1.833123192 | 3.85E-07 |
| hsa-miR-6851-3p | -1.834634717 | 1.04E-07 |
| hsa-miR-8075 | -1.836460547 | 1.69E-07 |
| hsa-miR-3191-5p | -1.844116231 | 2.77E-08 |
| hsa-miR-6716-3p | -1.847317942 | 1.40E-08 |
| hsa-miR-6769b-3p | -1.847764571 | 1.28E-07 |
| hsa-miR-6868-3p | -1.848169294 | 1.19E-08 |
| hsa-miR-6849-3p | -1.852721701 | 6.59E-08 |
| hsa-miR-3192-3p | -1.861577254 | 5.53E-08 |
| hsa-miR-6127 | -1.86337774 | 3.13E-07 |
| hsa-miR-4732-3p | -1.866723658 | 6.53E-09 |
| hsa-miR-5587-3p | -1.868578654 | 1.29E-07 |
| hsa-miR-3177-5p | -1.871394233 | 8.23E-08 |
| hsa-miR-4292 | -1.871508463 | 9.01E-08 |
| hsa-miR-4722-3p | -1.876165637 | 7.23E-07 |
| hsa-miR-6876-3p | -1.884394293 | 3.42E-10 |
| hsa-miR-6737-3p | -1.892466522 | 1.73E-08 |
| hsa-miR-6809-3p | -1.90222825 | 2.80E-08 |
| hsa-miR-6814-3p | -1.903297975 | 1.26E-09 |
| hsa-miR-6749-3p | -1.904606968 | 1.78E-10 |
| hsa-miR-4305 | -1.905320755 | 4.24E-12 |
| hsa-miR-30c-1-3p | -1.90630411 | 9.28E-10 |
| hsa-miR-133b | -1.907293212 | 5.78E-08 |
| hsa-miR-887-3p | -1.909374712 | 6.24E-31 |
| hsa-miR-4326 | -1.918864803 | 3.14E-10 |
| hsa-miR-3667-3p | -1.927331078 | 1.45E-12 |
| hsa-miR-6726-3p | -1.928067188 | 1.10E-08 |
| hsa-miR-615-3p | -1.928887333 | 2.23E-08 |
| hsa-miR-378f | -1.929892235 | 4.91E-09 |
| hsa-miR-1207-3p | -1.931465691 | 2.38E-09 |
| hsa-miR-3944-3p | -1.932336509 | 3.66E-08 |
| hsa-miR-9500 | -1.934980911 | 3.13E-08 |
| hsa-miR-6753-3p | -1.93531789 | 5.12E-09 |
| hsa-miR-885-5p | -1.938737228 | 4.56E-09 |
| hsa-miR-326 | -1.962694847 | 1.59E-08 |
| hsa-miR-4482-3p | -1.965288129 | 4.55E-08 |
| hsa-miR-1250-3p | -1.967661438 | 1.00E-09 |
| hsa-miR-6776-3p | -1.973372644 | 4.65E-08 |
| hsa-miR-5010-3p | -1.975744027 | 1.30E-09 |
| hsa-miR-659-3p | -1.979232493 | 4.92E-08 |
| hsa-miR-943 | -1.979571293 | 8.32E-10 |
| hsa-miR-6846-3p | -2.000660747 | 1.82E-08 |
| hsa-miR-6771-3p | -2.001423503 | 3.30E-09 |
| hsa-miR-5193 | -2.00691136 | 2.94E-08 |
| hsa-miR-887-5p | -2.006935964 | 2.19E-08 |
| hsa-miR-7152-5p | -2.014688984 | 1.21E-09 |
| hsa-miR-6795-3p | -2.015185644 | 4.86E-09 |
| hsa-miR-4329 | -2.022372941 | 9.98E-11 |
| hsa-miR-7848-3p | -2.030930715 | 2.31E-12 |
| hsa-miR-6826-3p | -2.037713996 | 1.29E-08 |
| hsa-miR-6836-5p | -2.038279674 | 4.46E-09 |
| hsa-miR-612 | -2.038513911 | 5.32E-09 |
| hsa-miR-2355-5p | -2.04697115 | 9.99E-09 |
| hsa-miR-4708-5p | -2.055091588 | 6.38E-09 |
| hsa-miR-1266-3p | -2.055628087 | 1.92E-11 |
| hsa-miR-937-3p | -2.055735755 | 2.37E-11 |
| hsa-miR-936 | -2.059052537 | 6.38E-10 |
| hsa-miR-548ab | -2.073189552 | 1.36E-18 |
| hsa-miR-764 | -2.082210078 | 2.49E-11 |
| hsa-miR-4804-3p | -2.085333607 | 2.75E-13 |
| hsa-miR-4314 | -2.08689648 | 1.64E-17 |
| hsa-miR-1296-5p | -2.091413108 | 1.93E-10 |
| hsa-miR-939-3p | -2.11389024 | 8.80E-10 |
| hsa-miR-6812-3p | -2.121402422 | 6.09E-09 |
| hsa-miR-629-3p | -2.122836113 | 7.08E-11 |
| hsa-miR-3945 | -2.126269836 | 2.28E-11 |
| hsa-miR-6827-3p | -2.126819542 | 7.73E-11 |
| hsa-miR-5006-5p | -2.127748985 | 5.90E-09 |
| hsa-miR-4506 | -2.128791325 | 2.90E-11 |
| hsa-miR-6894-3p | -2.129121307 | 3.92E-09 |
| hsa-miR-7110-3p | -2.143202468 | 3.59E-09 |
| hsa-miR-765 | -2.149605347 | 1.54E-09 |
| hsa-miR-503-3p | -2.151474186 | 1.12E-11 |
| hsa-miR-6886-3p | -2.160517842 | 4.95E-09 |
| hsa-miR-6861-3p | -2.16784572 | 8.02E-10 |
| hsa-miR-6878-3p | -2.16787778 | 1.01E-13 |
| hsa-miR-135a-3p | -2.172559609 | 1.86E-19 |
| hsa-miR-4717-3p | -2.176264531 | 1.20E-09 |
| hsa-miR-4780 | -2.186567465 | 1.06E-14 |
| hsa-miR-139-3p | -2.199316409 | 8.78E-10 |
| hsa-miR-6883-5p | -2.199631474 | 3.32E-17 |
| hsa-miR-2682-3p | -2.202163167 | 2.44E-10 |
| hsa-miR-6893-3p | -2.202236274 | 2.60E-10 |
| hsa-miR-6889-3p | -2.206360823 | 2.01E-10 |
| hsa-miR-6770-3p | -2.209569101 | 1.21E-09 |
| hsa-miR-26b-3p | -2.212562098 | 9.61E-11 |
| hsa-miR-6857-3p | -2.231151747 | 1.52E-12 |
| hsa-miR-6071 | -2.244211987 | 2.59E-21 |
| hsa-miR-3175 | -2.245805253 | 7.26E-10 |
| hsa-miR-4682 | -2.249467495 | 2.10E-13 |
| hsa-miR-6810-3p | -2.251109535 | 7.40E-11 |
| hsa-miR-6762-3p | -2.2677799 | 2.03E-12 |
| hsa-miR-92a-2-5p | -2.268513765 | 7.73E-36 |
| hsa-miR-4265 | -2.282216599 | 2.17E-10 |
| hsa-miR-6830-3p | -2.286401707 | 1.01E-10 |
| hsa-miR-3173-5p | -2.292978401 | 1.31E-12 |
| hsa-miR-642a-5p | -2.293916077 | 1.58E-10 |
| hsa-miR-6773-3p | -2.303046594 | 2.66E-14 |
| hsa-miR-1825 | -2.305573638 | 1.76E-10 |
| hsa-miR-7976 | -2.306735619 | 8.23E-20 |
| hsa-miR-6757-3p | -2.312174301 | 7.73E-16 |
| hsa-miR-6741-3p | -2.35723728 | 1.75E-11 |
| hsa-miR-6820-3p | -2.361791383 | 1.05E-11 |
| hsa-miR-2113 | -2.378517171 | 4.16E-26 |
| hsa-miR-6778-3p | -2.381247006 | 1.59E-13 |
| hsa-miR-3605-3p | -2.3893604 | 5.13E-14 |
| hsa-miR-6509-3p | -2.394695551 | 4.52E-22 |
| hsa-miR-6793-3p | -2.410544753 | 8.70E-12 |
| hsa-miR-1199-3p | -2.415730867 | 2.76E-12 |
| hsa-miR-6890-3p | -2.421042303 | 7.52E-12 |
| hsa-miR-6883-3p | -2.423189384 | 1.97E-13 |
| hsa-miR-6779-3p | -2.425029199 | 6.26E-12 |
| hsa-miR-1236-5p | -2.439675635 | 1.46E-11 |
| hsa-miR-6790-3p | -2.442753246 | 1.40E-12 |
| hsa-miR-1273h-5p | -2.454500926 | 1.07E-12 |
| hsa-miR-642b-5p | -2.464294288 | 4.28E-16 |
| hsa-miR-6740-3p | -2.474047478 | 6.30E-13 |
| hsa-miR-6884-3p | -2.476420024 | 1.19E-12 |
| hsa-miR-183-3p | -2.492155589 | 9.98E-18 |
| hsa-miR-3180-5p | -2.49453043 | 9.49E-13 |
| hsa-miR-1303 | -2.49756484 | 1.04E-11 |
| hsa-miR-6885-3p | -2.505934902 | 6.32E-13 |
| hsa-miR-602 | -2.521109274 | 3.59E-15 |
| hsa-miR-6775-3p | -2.541979755 | 1.06E-13 |
| hsa-miR-1304-3p | -2.542565868 | 6.15E-15 |
| hsa-miR-6787-3p | -2.548543784 | 4.45E-14 |
| hsa-miR-4269 | -2.554159087 | 2.99E-13 |
| hsa-miR-6780a-3p | -2.562353141 | 1.02E-27 |
| hsa-miR-6751-5p | -2.585108115 | 8.98E-14 |
| hsa-let-7b-3p | -2.589154437 | 1.10E-17 |
| hsa-miR-4313 | -2.598929705 | 1.10E-14 |
| hsa-miR-4435 | -2.61058928 | 1.44E-14 |
| hsa-miR-4748 | -2.632062384 | 9.00E-16 |
| hsa-miR-6511a-3p | -2.656540878 | 1.28E-13 |
| hsa-miR-3649 | -2.657198988 | 5.80E-19 |
| hsa-miR-6782-3p | -2.671088876 | 3.13E-14 |
| hsa-miR-4290 | -2.680263624 | 3.84E-15 |
| hsa-miR-6772-3p | -2.694589567 | 4.64E-21 |
| hsa-miR-4740-3p | -2.703442689 | 4.15E-14 |
| hsa-miR-5581-5p | -2.719382804 | 7.60E-16 |
| hsa-miR-7702 | -2.719504824 | 7.24E-19 |
| hsa-miR-4730 | -2.767123224 | 1.71E-111 |
| hsa-miR-6748-3p | -2.791798151 | 1.37E-16 |
| hsa-miR-6750-3p | -2.793349374 | 1.66E-14 |
| hsa-miR-542-5p | -2.811421114 | 4.28E-17 |
| hsa-miR-6794-3p | -2.819712297 | 1.06E-15 |
| hsa-miR-4479 | -2.836061131 | 1.22E-15 |
| hsa-miR-6133 | -2.849752878 | 1.56E-27 |
| hsa-miR-6786-3p | -2.849908272 | 1.89E-16 |
| hsa-miR-575 | -2.874720393 | 1.77E-26 |
| hsa-miR-6742-3p | -2.879751331 | 2.44E-20 |
| hsa-miR-2277-5p | -2.894625092 | 2.75E-19 |
| hsa-miR-6788-3p | -2.971827711 | 1.79E-20 |
| hsa-miR-6857-5p | -2.98909068 | 9.64E-15 |
| hsa-miR-4481 | -2.997177847 | 2.66E-15 |
| hsa-miR-1273c | -3.049136317 | 3.73E-24 |
| hsa-miR-935 | -3.06151788 | 2.76E-22 |
| hsa-miR-3194-5p | -3.066177453 | 2.08E-19 |
| hsa-miR-3713 | -3.133119499 | 6.05E-32 |
| hsa-miR-134-3p | -3.141966354 | 6.94E-26 |
| hsa-miR-873-3p | -3.150706449 | 4.38E-19 |
| hsa-miR-433-5p | -3.236016704 | 1.56E-27 |
| hsa-miR-3692-5p | -3.239798637 | 1.04E-34 |
| hsa-miR-3184-5p | -3.495620611 | 6.46E-40 |
| hsa-miR-30b-3p | -3.516945096 | 5.99E-27 |
| hsa-miR-1203 | -3.63554404 | 3.80E-30 |
| hsa-miR-6501-5p | -3.698694254 | 8.10E-33 |
| hsa-miR-508-5p | -3.774419711 | 8.22E-33 |
| hsa-miR-4276 | -3.868703946 | 3.41E-26 |
| hsa-miR-769-3p | -3.879560067 | 7.64E-34 |
| hsa-miR-4648 | -4.021529074 | 1.96E-30 |
| hsa-miR-125a-3p | -4.065122481 | 7.02E-32 |
| hsa-miR-125b-1-3p | -4.311967883 | 5.42E-51 |

**Table S6: List of Differentially Expressed miRNAs in prostate cancer from GSE112264 compared to negative PCa patients.**

| miRNA ID | logFC | adj.P.Val |
| --- | --- | --- |
| hsa-miR-1290 | 4.280134481 | 1.38E-252 |
| hsa-miR-17-3p | 4.095078897 | 3.34E-259 |
| hsa-miR-5100 | 3.354517132 | 0 |
| hsa-miR-495-5p | 2.989106303 | 7.13E-69 |
| hsa-miR-6839-5p | 2.95054599 | 6.13E-100 |
| hsa-miR-320d | 2.46500878 | 8.87E-59 |
| hsa-miR-6780a-5p | 2.44792301 | 2.11E-56 |
| hsa-miR-4440 | 2.246029968 | 4.82E-47 |
| hsa-miR-4754 | 2.189296374 | 3.31E-44 |
| hsa-miR-320c | 2.181439419 | 2.44E-56 |
| hsa-miR-6730-5p | 2.153051845 | 2.56E-47 |
| hsa-miR-1185-2-3p | 2.065543293 | 2.08E-89 |
| hsa-miR-4685-3p | 2.025346735 | 1.14E-38 |
| hsa-miR-5189-3p | 1.985616429 | 1.09E-36 |
| hsa-miR-4658 | 1.978630017 | 3.20E-36 |
| hsa-miR-1185-1-3p | 1.932811452 | 1.06E-94 |
| hsa-miR-422a | 1.918936298 | 8.39E-41 |
| hsa-miR-3132 | 1.911500452 | 6.28E-30 |
| hsa-miR-6776-5p | 1.894587487 | 2.82E-36 |
| hsa-miR-6754-3p | 1.881775712 | 3.22E-30 |
| hsa-miR-4652-5p | 1.864089726 | 7.88E-28 |
| hsa-miR-6751-3p | 1.861496764 | 1.19E-32 |
| hsa-miR-6761-3p | 1.851252117 | 5.90E-31 |
| hsa-miR-4695-3p | 1.843431507 | 3.94E-35 |
| hsa-miR-1273g-5p | 1.802181624 | 3.14E-28 |
| hsa-miR-5585-3p | 1.801152461 | 5.95E-22 |
| hsa-miR-4472 | 1.761363684 | 4.76E-32 |
| hsa-miR-3928-5p | 1.756707074 | 1.57E-27 |
| hsa-miR-6743-3p | 1.755702881 | 1.94E-37 |
| hsa-miR-598-5p | 1.715647656 | 1.93E-27 |
| hsa-miR-185-3p | 1.708714942 | 5.44E-34 |
| hsa-miR-3682-3p | 1.700664054 | 2.85E-25 |
| hsa-miR-519d-5p | 1.698383547 | 4.81E-32 |
| hsa-miR-6862-3p | 1.698017136 | 1.74E-29 |
| hsa-miR-451a | 1.696119044 | 2.65E-34 |
| hsa-miR-6747-3p | 1.694671227 | 3.74E-27 |
| hsa-miR-3944-5p | 1.693784512 | 1.04E-26 |
| hsa-miR-6859-5p | 1.689427662 | 1.01E-23 |
| hsa-miR-4633-5p | 1.678320999 | 2.42E-23 |
| hsa-miR-320b | 1.67805486 | 6.75E-70 |
| hsa-miR-6772-5p | 1.666527169 | 8.26E-27 |
| hsa-miR-4420 | 1.660822992 | 1.74E-22 |
| hsa-miR-3688-5p | 1.660746226 | 9.99E-24 |
| hsa-miR-520f-5p | 1.653405818 | 1.04E-26 |
| hsa-miR-1976 | 1.650221064 | 3.58E-31 |
| hsa-miR-504-3p | 1.639676039 | 8.91E-69 |
| hsa-miR-6876-5p | 1.615873073 | 3.14E-25 |
| hsa-miR-6793-5p | 1.61152017 | 8.27E-25 |
| hsa-miR-6801-5p | 1.606116602 | 3.37E-26 |
| hsa-miR-3646 | 1.606052146 | 1.21E-25 |
| hsa-miR-4713-5p | 1.599429015 | 7.32E-26 |
| hsa-miR-6856-5p | 1.595339022 | 2.40E-24 |
| hsa-miR-7109-3p | 1.593540675 | 3.60E-31 |
| hsa-miR-6734-5p | 1.581288291 | 1.57E-23 |
| hsa-miR-8085 | 1.578011621 | 2.01E-24 |
| hsa-miR-4769-5p | 1.577252996 | 1.92E-30 |
| hsa-miR-4701-3p | 1.574543097 | 5.27E-24 |
| hsa-miR-664b-3p | 1.573919377 | 4.25E-24 |
| hsa-miR-6831-5p | 1.572513314 | 1.31E-34 |
| hsa-miR-4738-3p | 1.558031327 | 6.53E-30 |
| hsa-miR-3173-5p | 1.543610373 | 8.73E-26 |
| hsa-miR-1296-3p | 1.542704217 | 1.20E-23 |
| hsa-miR-6735-3p | 1.542677873 | 5.54E-23 |
| hsa-miR-6894-3p | 1.542101673 | 3.08E-21 |
| hsa-miR-3622a-3p | 1.538664006 | 2.86E-23 |
| hsa-miR-3663-5p | 1.535789221 | 5.39E-23 |
| hsa-miR-3151-3p | 1.532102999 | 1.37E-23 |
| hsa-miR-6804-3p | 1.532004382 | 6.99E-24 |
| hsa-miR-6803-3p | 1.520527787 | 1.51E-23 |
| hsa-miR-3190-3p | 1.51717175 | 1.22E-22 |
| hsa-miR-3191-5p | 1.510247195 | 5.33E-24 |
| hsa-miR-5589-5p | 1.509493053 | 3.75E-22 |
| hsa-miR-4529-5p | 1.508142977 | 5.36E-21 |
| hsa-miR-6753-3p | 1.508083756 | 6.14E-24 |
| hsa-miR-6823-3p | 1.505735748 | 7.16E-21 |
| hsa-miR-6790-5p | 1.500836925 | 6.57E-26 |
| hsa-miR-3127-3p | -1.506140513 | 1.90E-22 |
| hsa-miR-1224-5p | -1.507641854 | 1.28E-38 |
| hsa-miR-133a-3p | -1.516566177 | 1.13E-20 |
| hsa-miR-3162-3p | -1.532297852 | 2.93E-27 |
| hsa-miR-302b-5p | -1.541078981 | 7.16E-21 |
| hsa-miR-6853-3p | -1.542387858 | 3.45E-32 |
| hsa-miR-6812-3p | -1.543213515 | 6.45E-21 |
| hsa-miR-1283 | -1.550689968 | 5.16E-35 |
| hsa-miR-4655-3p | -1.554364621 | 1.34E-15 |
| hsa-miR-323a-3p | -1.589753793 | 3.18E-26 |
| hsa-miR-548s | -1.625590359 | 1.78E-20 |
| hsa-miR-548f-3p | -1.643219362 | 5.33E-24 |
| hsa-miR-136-3p | -1.645349514 | 9.52E-32 |
| hsa-miR-548o-3p | -1.684546391 | 4.46E-34 |
| hsa-miR-29b-2-5p | -1.685638375 | 3.68E-23 |
| hsa-miR-6084 | -1.690214658 | 3.35E-30 |
| hsa-miR-1302 | -1.695276443 | 4.71E-27 |
| hsa-miR-195-3p | -1.703122284 | 4.91E-23 |
| hsa-miR-192-3p | -1.723604685 | 6.42E-25 |
| hsa-miR-21-5p | -1.732705632 | 1.83E-24 |
| hsa-miR-409-5p | -1.752872754 | 4.43E-25 |
| hsa-miR-3142 | -1.754565959 | 2.87E-31 |
| hsa-miR-6070 | -1.825142094 | 3.94E-35 |
| hsa-let-7f-1-3p | -1.830191337 | 6.65E-31 |
| hsa-miR-767-3p | -1.849153339 | 1.09E-35 |
| hsa-miR-331-3p | -1.883029788 | 3.39E-30 |
| hsa-miR-640 | -1.893114501 | 1.09E-33 |
| hsa-miR-518e-3p | -1.898731118 | 6.24E-27 |
| hsa-miR-548ah-3p | -1.934085853 | 6.76E-31 |
| hsa-miR-425-3p | -1.97269501 | 3.13E-34 |
| hsa-miR-4292 | -1.978295854 | 8.55E-36 |
| hsa-miR-129-1-3p | -2.024459921 | 3.96E-38 |
| hsa-miR-944 | -2.044580609 | 8.39E-41 |
| hsa-miR-4727-5p | -2.046755183 | 1.04E-41 |
| hsa-miR-106a-3p | -2.130265342 | 5.32E-34 |
| hsa-miR-6869-3p | -2.265057492 | 2.49E-42 |
| hsa-miR-515-5p | -2.398059802 | 3.80E-45 |
| hsa-miR-1227-3p | -2.487098902 | 1.14E-52 |
| hsa-miR-4313 | -2.543423592 | 7.32E-60 |
| hsa-miR-3181 | -2.743363677 | 3.22E-73 |
| hsa-miR-31-3p | -2.91394025 | 7.08E-66 |
| hsa-miR-941 | -2.928513952 | 2.40E-78 |
| hsa-miR-516a-5p | -3.00297482 | 2.53E-81 |
| hsa-miR-646 | -3.231486803 | 6.21E-75 |
| hsa-miR-4456 | -6.150324786 | 0 |

Table S7: List of Differentially Expressed miRNAs in prostate cancer from GSE112264 compared to Bladder cancer.

| miRNA ID | logFC | adj.P.Val |
| --- | --- | --- |
| hsa-miR-8085 | 2.850030833 | 9.25E-19 |
| hsa-miR-6793-5p | 2.305994307 | 3.27E-12 |
| hsa-miR-4419a | 2.299675267 | 2.53E-13 |
| hsa-miR-1587 | 2.162637851 | 1.61E-10 |
| hsa-miR-6780a-5p | 2.157723299 | 2.53E-11 |
| hsa-miR-4440 | 2.093499278 | 1.73E-10 |
| hsa-miR-6796-5p | 2.054279936 | 5.77E-10 |
| hsa-miR-3184-3p | 1.919350912 | 6.07E-08 |
| hsa-miR-6165 | 1.895934231 | 6.97E-19 |
| hsa-miR-6792-3p | 1.855290041 | 1.23E-08 |
| hsa-miR-8485 | 1.84631482 | 1.15E-10 |
| hsa-miR-6859-5p | 1.839352315 | 4.77E-07 |
| hsa-miR-6890-5p | 1.837306461 | 1.98E-07 |
| hsa-miR-4481 | 1.762660967 | 3.12E-06 |
| hsa-miR-564 | 1.739198819 | 1.22E-08 |
| hsa-miR-7846-3p | 1.72898218 | 5.12E-15 |
| hsa-miR-6827-5p | 1.715249594 | 3.29E-08 |
| hsa-miR-6849-5p | 1.690523514 | 1.12E-06 |
| hsa-miR-6086 | 1.650649188 | 5.77E-10 |
| hsa-miR-574-3p | 1.645568998 | 8.81E-09 |
| hsa-miR-4478 | 1.629445808 | 3.65E-07 |
| hsa-miR-4268 | 1.618145492 | 1.94E-06 |
| hsa-miR-6895-5p | 1.615306355 | 4.72E-08 |
| hsa-miR-6731-5p | 1.593338744 | 3.75E-07 |
| hsa-miR-6127 | 1.59214027 | 9.65E-06 |
| hsa-miR-6753-5p | 1.517612949 | 4.12E-10 |
| hsa-miR-7160-5p | 1.516062133 | 7.57E-06 |
| hsa-miR-4789-5p | -1.60170382 | 8.53E-05 |
| hsa-miR-4652-5p | -2.27178577 | 3.66E-10 |

**Table S8: List of Differentially Expressed miRNAs in Prostate Cancer from GSE159177 and GSE138740**

| miRNA ID | logFC | adj.P.Val |
| --- | --- | --- |
| hsa-miR-5000-5p | 2.793707538 | 0.001358313 |
| hsa-let-7i-5p | 2.445880259 | 2.13E-09 |
| hsa-miR-6797-3p | 2.227373432 | 0.003635349 |
| hsa-miR-1825 | 2.1783732 | 0.000190189 |
| hsa-miR-6836-3p | 2.069643028 | 0.025038776 |
| hsa-miR-4701-5p | 1.674524835 | 5.45E-05 |
| hsa-miR-532-3p | 1.511123653 | 0.040810822 |
| hsa-miR-6511b-3p | 1.496044227 | 0.00450443 |
| hsa-miR-25-3p | 1.445539959 | 9.88E-27 |
| hsa-miR-4743-5p | 1.433300903 | 0.000730204 |
| hsa-miR-4518 | 1.37242825 | 0.000270678 |
| hsa-mir-506 | 1.360448642 | 2.23E-09 |
| hsa-miR-6766-3p | 1.348159902 | 0.035116055 |
| hsa-mir-4638 | 1.346161307 | 1.32E-11 |
| hsa-mir-5094 | 1.338576655 | 1.05E-08 |
| hsa-miR-605-3p | 1.296046561 | 2.12E-11 |
| hsa-miR-6732-3p | 1.279633684 | 0.000150997 |
| hsa-miR-16-1-3p | 1.279602523 | 6.65E-09 |
| hsa-miR-29a-5p | 1.268763068 | 6.89E-21 |
| hsa-miR-1224-3p | 1.180094546 | 0.044103584 |
| hsa-mir-4518 | 1.176941129 | 8.91E-08 |
| hsa-miR-6841-3p | 1.170868299 | 1.69E-12 |
| hsa-mir-185 | 1.15234348 | 2.67E-05 |
| hsa-miR-7150 | 1.149620486 | 0.01289792 |
| hsa-mir-4329 | 1.136121047 | 0.04042314 |
| hsa-miR-4654 | 1.134438307 | 0.004155597 |
| hsa-miR-660-5p | 1.131194867 | 9.42E-07 |
| hsa-miR-6515-3p | 1.123435248 | 4.28E-05 |
| hsa-miR-6861-3p | 1.112307534 | 0.001005298 |
| hsa-mir-143 | 1.103239534 | 7.61E-10 |
| hsa-miR-548aj-3p | 1.059113727 | 0.014321931 |
| hsa-mir-6808 | 1.0500548 | 0.000603717 |
| hsa-miR-3940-3p | 1.048538542 | 0.002307551 |
| hsa-miR-662 | 1.033064579 | 1.65E-05 |
| hsa-mir-4271 | 1.014130594 | 4.55E-11 |
| hsa-miR-6787-5p | -1.396262955 | 0.03127803 |
| hsa-miR-6794-5p | -1.436228165 | 0.025606049 |
| hsa-miR-4467 | -2.544669141 | 0.009004831 |

**Table S9: List of Differentially Expressed miRNAs in high-grade prostate cancer compared to the Control group from GSE86474.**

| miRNA ID | logFC | adj.P.Val |
| --- | --- | --- |
| hsa-miR-223-3p | 6.27857724 | 9.36E-09 |
| hsa-miR-451a | 4.533989633 | 8.63E-06 |
| hsa-miR-142-3p | 4.160989404 | 9.26E-06 |
| hsa-miR-320e | 2.972567926 | 0.000159819 |
| hsa-miR-4516 | 2.917183394 | 1.45E-07 |
| hsa-miR-205-5p | 2.827723116 | 7.07E-05 |
| hsa-miR-630 | 2.120066407 | 5.59E-05 |
| hsa-miR-204-5p | 1.995204717 | 9.88E-05 |
| hsa-miR-1972 | 1.944187559 | 9.33E-05 |
| hsa-miR-150-5p | 1.823323901 | 0.006231592 |
| hsa-miR-575 | 1.524255693 | 0.0015446 |
| hsa-miR-2116-5p | -1.217775519 | 0.00064615 |
| hsa-miR-30c-5p | -1.223368315 | 0.005353465 |
| hsa-miR-744-5p | -1.233434908 | 0.000170026 |
| hsa-miR-644a | -1.242662455 | 4.80E-05 |
| hsa-miR-345-5p | -1.258081434 | 5.27E-05 |
| hsa-miR-941 | -1.276859536 | 0.000147664 |
| hsa-miR-28-3p | -1.341695086 | 8.55E-05 |
| hsa-miR-1260b | -1.345742388 | 0.000103096 |
| hsa-miR-454-3p | -1.345789941 | 6.62E-05 |
| hsa-miR-335-5p | -1.354601466 | 2.09E-05 |
| hsa-miR-532-5p | -1.361189051 | 0.000138724 |
| hsa-miR-574-3p | -1.373833959 | 0.000107716 |
| hsa-miR-340-5p | -1.399088559 | 0.000365373 |
| hsa-miR-4455 | -1.40068897 | 7.32E-07 |
| hsa-miR-185-5p | -1.421386584 | 0.001259759 |
| hsa-miR-193a-3p | -1.431147704 | 2.23E-06 |
| hsa-miR-33a-5p | -1.449907303 | 4.05E-06 |
| hsa-miR-484 | -1.469434692 | 3.13E-06 |
| hsa-miR-27b-3p | -1.4762471 | 0.008735378 |
| hsa-miR-34c-5p | -1.551711927 | 5.27E-08 |
| hsa-miR-362-3p | -1.56263792 | 0.000298545 |
| hsa-miR-26a-5p | -1.582643377 | 0.005494695 |
| hsa-miR-1247-5p | -1.583906737 | 3.80E-05 |
| hsa-miR-590-5p | -1.642082206 | 1.45E-07 |
| hsa-miR-22-3p | -1.642188743 | 0.003280427 |
| hsa-miR-15a-5p | -1.654753342 | 0.037265537 |
| hsa-miR-222-3p | -1.65547916 | 0.00064615 |
| hsa-miR-1206 | -1.702774737 | 4.41E-09 |
| hsa-miR-196b-5p | -1.703779848 | 3.14E-06 |
| hsa-miR-34a-5p | -1.710360906 | 0.001251775 |
| hsa-miR-7-5p | -1.711398244 | 6.83E-08 |
| hsa-miR-218-5p | -1.729808991 | 7.01E-10 |
| hsa-miR-27a-3p | -1.757790346 | 4.30E-07 |
| hsa-miR-148b-3p | -1.797580715 | 2.50E-05 |
| hsa-miR-497-5p | -1.835871863 | 2.55E-07 |
| hsa-miR-92b-3p | -1.849135445 | 4.41E-09 |
| hsa-miR-193b-3p | -1.906138666 | 6.97E-08 |
| hsa-miR-423-5p | -1.921672068 | 5.86E-05 |
| hsa-miR-186-5p | -1.932832733 | 1.15E-07 |
| hsa-miR-29c-3p | -1.999420819 | 3.43E-05 |
| hsa-miR-429 | -2.071922739 | 6.98E-08 |
| hsa-miR-141-3p | -2.079024129 | 0.000217134 |
| hsa-miR-361-5p | -2.14070794 | 6.38E-07 |
| hsa-miR-191-5p | -2.173616038 | 0.003034781 |
| hsa-miR-126-3p | -2.211017979 | 1.05E-09 |
| hsa-miR-151a-5p | -2.221907185 | 5.70E-10 |
| hsa-miR-197-3p | -2.245337918 | 1.14E-11 |
| hsa-miR-25-3p | -2.297318183 | 0.000431692 |
| hsa-let-7g-5p | -2.331851793 | 0.001048171 |
| hsa-miR-130b-3p | -2.342585491 | 3.62E-09 |
| hsa-miR-183-5p | -2.343198818 | 9.90E-13 |
| hsa-miR-374b-5p | -2.361400282 | 5.98E-09 |
| hsa-miR-148a-3p | -2.381380699 | 0.000145812 |
| hsa-miR-200c-3p | -2.396815177 | 0.000725638 |
| hsa-miR-301a-3p | -2.407155482 | 7.52E-11 |
| hsa-miR-151a-3p | -2.428125358 | 3.99E-11 |
| hsa-miR-296-5p | -2.45860459 | 1.74E-14 |
| hsa-miR-31-5p | -2.512658711 | 1.14E-11 |
| hsa-miR-107 | -2.542383137 | 1.78E-08 |
| hsa-miR-374a-5p | -2.559813233 | 2.30E-06 |
| hsa-let-7d-5p | -2.699764651 | 1.45E-06 |
| hsa-miR-425-5p | -2.735259982 | 1.17E-06 |
| hsa-miR-324-5p | -2.7747744 | 3.08E-10 |
| hsa-miR-15b-5p | -2.831290538 | 7.34E-05 |
| hsa-miR-96-5p | -2.849873698 | 6.83E-20 |
| hsa-miR-16-5p | -2.860586279 | 0.000294639 |
| hsa-miR-9-5p | -2.900547616 | 4.68E-16 |
| hsa-miR-423-3p | -2.909463619 | 6.57E-11 |
| hsa-miR-30b-5p | -2.920262256 | 1.53E-10 |
| hsa-miR-130a-3p | -2.947243846 | 2.28E-10 |
| hsa-miR-32-5p | -2.973647321 | 1.52E-11 |
| hsa-miR-18a-5p | -2.979491359 | 4.68E-16 |
| hsa-miR-1260a | -2.989602742 | 8.95E-09 |
| hsa-miR-93-5p | -3.033929377 | 1.98E-06 |
| hsa-miR-24-3p | -3.147898812 | 1.18E-06 |
| hsa-let-7f-5p | -3.222742313 | 1.78E-08 |
| hsa-let-7b-5p | -3.272080264 | 3.22E-07 |
| hsa-miR-106b-5p | -3.278725356 | 5.18E-11 |
| hsa-let-7i-5p | -3.313268118 | 8.95E-09 |
| hsa-miR-331-3p | -3.331308015 | 2.88E-15 |
| hsa-let-7e-5p | -3.422722024 | 9.78E-13 |
| hsa-miR-99b-5p | -3.470215665 | 3.89E-15 |
| hsa-miR-125a-5p | -3.530742287 | 5.48E-09 |
| hsa-miR-29a-3p | -3.533817634 | 2.31E-09 |
| hsa-miR-19a-3p | -3.539540454 | 5.18E-11 |
| hsa-miR-92a-3p | -3.651124368 | 3.05E-18 |
| hsa-miR-106a-5p+hsa-miR-17-5p | -3.686918299 | 1.32E-11 |
| hsa-miR-20a-5p+hsa-miR-20b-5p | -3.714852475 | 3.11E-09 |
| hsa-miR-19b-3p | -3.722566423 | 5.70E-10 |
| hsa-miR-182-5p | -3.751085176 | 1.25E-14 |
| hsa-miR-29b-3p | -3.978471432 | 1.41E-08 |
| hsa-miR-221-3p | -4.04394415 | 1.17E-17 |
| hsa-miR-99a-5p | -4.795802237 | 2.37E-15 |
| hsa-miR-100-5p | -5.546141949 | 1.38E-33 |

**Table S10: List of Differentially Expressed miRNAs in low-grade prostate cancer compared to the Control group from GSE86474.**

| miRNA ID | logFC | adj.P.Val |
| --- | --- | --- |
| hsa-miR-223-3p | 5.062685784 | 1.46E-06 |
| hsa-miR-142-3p | 3.14918718 | 0.000573727 |
| hsa-miR-451a | 3.145186899 | 0.001569164 |
| hsa-miR-4516 | 2.562743024 | 1.66E-06 |
| hsa-miR-320e | 2.114246706 | 0.00673457 |
| hsa-miR-205-5p | 2.10945943 | 0.002363291 |
| hsa-miR-630 | 1.826370232 | 0.000322466 |
| hsa-miR-204-5p | 1.573192503 | 0.001569164 |
| hsa-miR-1972 | 1.096368224 | 0.032181144 |
| hsa-miR-644a | -1.004262408 | 0.000703345 |
| hsa-miR-335-5p | -1.171217485 | 0.000133633 |
| hsa-miR-30c-5p | -1.306947497 | 0.001569164 |
| hsa-miR-193a-3p | -1.346729292 | 3.92E-06 |
| hsa-miR-574-3p | -1.347558283 | 7.29E-05 |
| hsa-miR-345-5p | -1.361780748 | 4.55E-06 |
| hsa-miR-744-5p | -1.389100275 | 9.20E-06 |
| hsa-miR-941 | -1.444307804 | 6.79E-06 |
| hsa-miR-33a-5p | -1.449836254 | 1.66E-06 |
| hsa-miR-4455 | -1.463310376 | 7.47E-08 |
| hsa-miR-532-5p | -1.468142612 | 1.66E-05 |
| hsa-miR-28-3p | -1.469001693 | 6.62E-06 |
| hsa-miR-1247-5p | -1.486150388 | 5.50E-05 |
| hsa-miR-2116-5p | -1.490767408 | 1.01E-05 |
| hsa-miR-484 | -1.511019261 | 6.39E-07 |
| hsa-miR-340-5p | -1.555476519 | 3.12E-05 |
| hsa-miR-590-5p | -1.644722745 | 4.49E-08 |
| hsa-miR-454-3p | -1.654298458 | 2.59E-07 |
| hsa-miR-1260b | -1.775301721 | 7.42E-08 |
| hsa-miR-26a-5p | -1.785797894 | 0.000826277 |
| hsa-miR-196b-5p | -1.795205594 | 3.39E-07 |
| hsa-miR-1206 | -1.798337549 | 1.38E-10 |
| hsa-miR-92b-3p | -1.802929354 | 2.58E-09 |
| hsa-miR-218-5p | -1.803312493 | 2.68E-11 |
| hsa-miR-362-3p | -1.867557698 | 5.77E-06 |
| hsa-miR-27b-3p | -1.881849365 | 0.000294821 |
| hsa-miR-185-5p | -1.890386014 | 5.34E-06 |
| hsa-miR-34c-5p | -1.897282097 | 9.16E-12 |
| hsa-miR-497-5p | -1.914459049 | 2.35E-08 |
| hsa-miR-193b-3p | -1.953568511 | 9.58E-09 |
| hsa-miR-7-5p | -1.994886562 | 8.85E-11 |
| hsa-miR-23a-3p | -1.999748097 | 0.002876338 |
| hsa-miR-27a-3p | -2.043587251 | 1.24E-09 |
| hsa-miR-186-5p | -2.052421648 | 5.15E-09 |
| hsa-miR-29c-3p | -2.074559295 | 6.79E-06 |
| hsa-miR-22-3p | -2.165800851 | 3.15E-05 |
| hsa-miR-148b-3p | -2.17723755 | 8.46E-08 |
| hsa-miR-34a-5p | -2.177305474 | 1.27E-05 |
| hsa-miR-197-3p | -2.19080957 | 4.87E-12 |
| hsa-miR-222-3p | -2.209504774 | 1.46E-06 |
| hsa-miR-429 | -2.24162246 | 1.60E-09 |
| hsa-miR-423-5p | -2.259086873 | 7.10E-07 |
| hsa-miR-141-3p | -2.288353996 | 1.99E-05 |
| hsa-miR-15a-5p | -2.288916031 | 0.001248173 |
| hsa-miR-151a-5p | -2.353413959 | 1.07E-11 |
| hsa-miR-31-5p | -2.407853708 | 9.86E-12 |
| hsa-miR-296-5p | -2.463929361 | 2.23E-15 |
| hsa-miR-148a-3p | -2.492274426 | 3.15E-05 |
| hsa-miR-130b-3p | -2.496337821 | 7.92E-11 |
| hsa-miR-361-5p | -2.526453111 | 1.21E-09 |
| hsa-miR-200c-3p | -2.545349588 | 0.000162419 |
| hsa-miR-126-3p | -2.581713761 | 3.72E-13 |
| hsa-miR-374b-5p | -2.65486647 | 1.56E-11 |
| hsa-let-7g-5p | -2.692985894 | 6.09E-05 |
| hsa-miR-107 | -2.696375566 | 6.68E-10 |
| hsa-miR-191-5p | -2.701240768 | 7.94E-05 |
| hsa-miR-183-5p | -2.732747721 | 2.69E-17 |
| hsa-miR-30b-5p | -2.734553518 | 3.73E-10 |
| hsa-miR-301a-3p | -2.748822732 | 3.22E-14 |
| hsa-miR-151a-3p | -2.768901726 | 1.30E-14 |
| hsa-miR-374a-5p | -2.795512215 | 7.94E-08 |
| hsa-miR-25-3p | -2.977951146 | 1.46E-06 |
| hsa-miR-324-5p | -3.021189657 | 1.87E-12 |
| hsa-miR-1260a | -3.032514456 | 1.47E-09 |
| hsa-miR-96-5p | -3.03547087 | 3.70E-23 |
| hsa-miR-423-3p | -3.057876717 | 1.63E-12 |
| hsa-let-7d-5p | -3.065477552 | 1.32E-08 |
| hsa-miR-425-5p | -3.0819054 | 1.36E-08 |
| hsa-miR-9-5p | -3.083913347 | 8.37E-19 |
| hsa-miR-32-5p | -3.140869527 | 2.23E-13 |
| hsa-miR-18a-5p | -3.204489993 | 5.12E-19 |
| hsa-miR-130a-3p | -3.254326729 | 6.39E-13 |
| hsa-miR-16-5p | -3.342658076 | 8.59E-06 |
| hsa-let-7f-5p | -3.343085326 | 1.47E-09 |
| hsa-miR-15b-5p | -3.38598756 | 6.65E-07 |
| hsa-miR-93-5p | -3.426839522 | 2.31E-08 |
| hsa-miR-106b-5p | -3.515650356 | 4.32E-13 |
| hsa-miR-331-3p | -3.522869064 | 1.34E-17 |
| hsa-miR-19a-3p | -3.651400347 | 2.58E-12 |
| hsa-miR-24-3p | -3.656182569 | 5.08E-09 |
| hsa-let-7e-5p | -3.658539381 | 4.15E-15 |
| hsa-let-7b-5p | -3.672304102 | 2.72E-09 |
| hsa-let-7i-5p | -3.781442972 | 1.46E-11 |
| hsa-miR-106a-5p+hsa-miR-17-5p | -3.791815671 | 6.31E-13 |
| hsa-miR-99b-5p | -3.796709747 | 2.03E-18 |
| hsa-miR-20a-5p+hsa-miR-20b-5p | -3.828746251 | 2.40E-10 |
| hsa-miR-92a-3p | -3.898619002 | 2.15E-21 |
| hsa-miR-19b-3p | -3.96617084 | 9.50E-12 |
| hsa-miR-182-5p | -4.004942203 | 3.75E-17 |
| hsa-miR-29a-3p | -4.006144754 | 3.37E-12 |
| hsa-miR-125a-5p | -4.036674141 | 9.16E-12 |
| hsa-miR-29b-3p | -4.200731776 | 5.95E-10 |
| hsa-miR-221-3p | -4.44846385 | 1.39E-21 |
| hsa-miR-99a-5p | -4.985852372 | 2.16E-17 |
| hsa-miR-100-5p | -5.595788025 | 1.61E-35 |

**Table S11: miRNAs that are differentially expressed between Gleason high - NA**

| miRNA Name | basement | log2FoldChange | lfcSE | stat | pvalue | padj |
| --- | --- | --- | --- | --- | --- | --- |
| hsa-miR-200c-3p | 4.20231214 | -2.991778723 | 0.500328 | -5.97963 | 2.24E-09 | 5.93E-06 |
| hsa-miR-98-5p | 2.63583815 | -2.536940482 | 0.452435 | -5.60731 | 2.06E-08 | 2.72E-05 |
| hsa-let-7e-5p | 3.32369942 | -2.287162387 | 0.433303 | -5.27843 | 1.30E-07 | 0.000115 |
| hsa-miR-23b-3p | 2.66473988 | 2.29067632 | 0.449193 | 5.099542 | 3.40E-07 | 0.000181 |
| hsa-miR-24-3p | 2.14450867 | -2.265344124 | 0.442371 | -5.12092 | 3.04E-07 | 0.000181 |
| hsa-miR-200b-3p | 30.1213873 | -3.246115457 | 0.703563 | -4.61383 | 3.95E-06 | 0.001747 |
| hsa-miR-30a-3p | 3.39306358 | -2.295932034 | 0.507438 | -4.52455 | 6.05E-06 | 0.002293 |
| hsa-let-7g-5p | 4.75144509 | -2.247926972 | 0.511797 | -4.39222 | 1.12E-05 | 0.003719 |
| hsa-miR-125b-5p | 1.97687861 | -1.859821999 | 0.425978 | -4.366 | 1.27E-05 | 0.003729 |
| hsa-miR-191-5p | 2.38150289 | -1.999999566 | 0.460809 | -4.3402 | 1.42E-05 | 0.003775 |
| hsa-miR-10a-5p | 16.5433526 | -2.816691118 | 0.652149 | -4.31909 | 1.57E-05 | 0.003777 |
| hsa-miR-92a-3p | 26.2427746 | -2.609441217 | 0.66396 | -3.93012 | 8.49E-05 | 0.018764 |
| hsa-miR-342-3p | 2.51445087 | -1.680119598 | 0.432583 | -3.88392 | 0.000103 | 0.01947 |
| hsa-miR-484 | 1.68208092 | -1.530514471 | 0.392779 | -3.89663 | 9.75E-05 | 0.01947 |
| hsa-miR-22-3p | 16.5953757 | -2.382468434 | 0.630079 | -3.78122 | 0.000156 | 0.027591 |
| hsa-miR-200a-5p | 3.27745665 | -1.750427594 | 0.466748 | -3.75026 | 0.000177 | 0.02928 |
| hsa-miR-186-5p | 1.54913295 | -1.499232384 | 0.402559 | -3.72425 | 0.000196 | 0.030559 |
| hsa-miR-615-3p | 1.19075145 | 1.132450084 | 0.314612 | 3.599515 | 0.000319 | 0.046971 |

**Table S12: miRNAs that are differentially expressed between Gleason low - NA**

| miRNA Name | baseMean | log2FoldChange | lfcSE | stat | pvalue | padj |
| --- | --- | --- | --- | --- | --- | --- |
| hsa-let-7e-5p | 3.32369942 | -2.606381358 | 0.301097 | -8.65628 | 4.87E-18 | 1.29E-14 |
| hsa-miR-98-5p | 2.63583815 | -2.52721115 | 0.300912 | -8.39849 | 4.52E-17 | 6.00E-14 |
| hsa-miR-24-3p | 2.14450867 | -2.265344467 | 0.292551 | -7.74342 | 9.68E-15 | 8.55E-12 |
| hsa-miR-200c-3p | 4.20231214 | -2.504709899 | 0.332897 | -7.52397 | 5.31E-14 | 3.52E-11 |
| hsa-miR-342-3p | 2.51445087 | -2.240782807 | 0.307878 | -7.27815 | 3.38E-13 | 1.80E-10 |
| hsa-miR-146a-5p | 2.41618497 | -1.904462752 | 0.29133 | -6.53714 | 6.27E-11 | 2.77E-08 |
| hsa-miR-200a-3p | 2.34104046 | -1.741660274 | 0.287373 | -6.06063 | 1.36E-09 | 5.14E-07 |
| hsa-miR-125b-5p | 1.97687861 | -1.589307927 | 0.276524 | -5.74746 | 9.06E-09 | 3.00E-06 |
| hsa-miR-186-5p | 1.54913295 | -1.499232572 | 0.266572 | -5.62412 | 1.86E-08 | 5.49E-06 |
| hsa-miR-484 | 1.68208093 | -1.399930558 | 0.256018 | -5.4681 | 4.55E-08 | 1.21E-05 |
| hsa-miR-221-3p | 1.74566474 | -1.49832168 | 0.282864 | -5.29696 | 1.18E-07 | 2.84E-05 |
| hsa-miR-200a-5p | 3.27745665 | -1.590096307 | 0.322088 | -4.93684 | 7.94E-07 | 0.000175 |
| hsa-miR-8083 | 1.97687861 | 1.452512155 | 0.321432 | 4.518881 | 6.22E-06 | 0.001268 |
| hsa-let-7g-5p | 4.75144509 | -1.546880336 | 0.350375 | -4.41492 | 1.01E-05 | 0.001914 |
| hsa-miR-320c | 1.42774567 | -1.029880533 | 0.23694 | -4.34658 | 1.38E-05 | 0.002445 |
| hsa-miR-3184-3p | 1.3583815 | -1.050862735 | 0.252004 | -4.17002 | 3.05E-05 | 0.004487 |
| hsa-miR-423-5p | 1.3583815 | -1.050862735 | 0.252004 | -4.17002 | 3.05E-05 | 0.004487 |
| hsa-miR-9985 | 3.30057804 | -1.331635271 | 0.318057 | -4.18678 | 2.83E-05 | 0.004487 |
| hsa-miR-191-5p | 2.38150289 | -1.223423987 | 0.294526 | -4.15387 | 3.27E-05 | 0.004563 |
| hsa-miR-23b-3p | 2.66473988 | 1.389318211 | 0.349285 | 3.977608 | 6.96E-05 | 0.009231 |
| hsa-miR-137-3p | 1.66473988 | -0.92644681 | 0.24101 | -3.84402 | 0.000121 | 0.015285 |

**
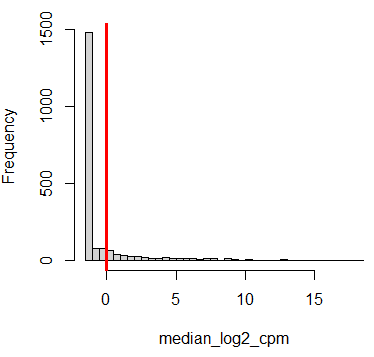
**

**Figure S2. Histogram of median log2-transformed counts per million (CPM) for miRNAs in the dataset**. The red vertical line represents the expression cutoff threshold used to filter out low-expressing miRNAs. miRNAs with median log2 CPM values greater than the threshold were retained for further analysis.

**Table S13: miRNAs that are differentially expressed between primary tumors and normal solid tissues using edgeR package**

| miRNA Name | logFC | logCPM | LR | PValue | adjPValue |
| --- | --- | --- | --- | --- | --- |
| hsa-miR-891a-5p | 4.600610303 | 4.031653064 | 732.3426988 | 2.77E-161 | 1.26E-158 |
| hsa-miR-93-5p | -1.81321238 | 11.70440139 | 237.6075527 | 1.31E-53 | 2.89E-51 |
| hsa-miR-1251-5p | 2.66250517 | 2.244254268 | 236.8546412 | 1.91E-53 | 2.89E-51 |
| hsa-miR-204-5p | 1.729477872 | 4.661282206 | 203.763612 | 3.15E-46 | 3.58E-44 |
| hsa-miR-27b-3p | 0.947348584 | 10.69415034 | 200.0243853 | 2.06E-45 | 1.87E-43 |
| hsa-miR-221-3p | 1.437498609 | 7.796558043 | 185.2439449 | 3.47E-42 | 2.63E-40 |
| hsa-miR-145-3p | 1.163170175 | 7.226089535 | 178.4767202 | 1.04E-40 | 6.76E-39 |
| hsa-miR-23b-3p | 0.893346992 | 10.59424875 | 172.1980577 | 2.45E-39 | 1.24E-37 |
| hsa-miR-200c-3p | -1.6264379 | 13.5527329 | 172.3725575 | 2.24E-39 | 1.24E-37 |
| hsa-miR-17-5p | -1.5463449 | 8.110150327 | 171.2159249 | 4.01E-39 | 1.82E-37 |
| hsa-miR-889-3p | 1.169188809 | 3.894666232 | 166.4799154 | 4.35E-38 | 1.79E-36 |
| hsa-miR-96-5p | -2.00277354 | 4.831780161 | 165.2925099 | 7.90E-38 | 2.99E-36 |
| hsa-miR-148a-3p | -1.46961223 | 15.86487238 | 160.5522985 | 8.57E-37 | 2.99E-35 |
| hsa-miR-136-3p | 1.420276904 | 3.858980583 | 159.3827996 | 1.54E-36 | 5.01E-35 |
| hsa-miR-20a-5p | -1.7266254 | 7.918662145 | 155.7762602 | 9.47E-36 | 2.87E-34 |
| hsa-miR-92a-3p | -1.16779519 | 12.82231661 | 148.2416093 | 4.20E-34 | 1.19E-32 |
| hsa-miR-652-3p | 1.100995447 | 3.651261536 | 146.5658863 | 9.76E-34 | 2.61E-32 |
| hsa-miR-375 | -1.93704775 | 16.57123064 | 145.2163611 | 1.93E-33 | 4.86E-32 |
| hsa-miR-187-3p | 2.335300205 | 3.630215823 | 140.983723 | 1.62E-32 | 3.88E-31 |
| hsa-miR-99b-3p | 0.904690225 | 4.703246728 | 137.9718014 | 7.39E-32 | 1.68E-30 |
| hsa-miR-182-5p | -1.93529474 | 14.16967939 | 137.2886073 | 1.04E-31 | 2.25E-30 |
| hsa-miR-25-3p | -1.10191393 | 12.61378067 | 135.926592 | 2.07E-31 | 4.27E-30 |
| hsa-miR-153-5p | -2.47784379 | 5.077295157 | 129.0539154 | 6.60E-30 | 1.30E-28 |
| hsa-miR-378c | 1.095578143 | 3.10372462 | 123.8348546 | 9.16E-29 | 1.73E-27 |
| hsa-miR-19b-3p | -1.37216607 | 7.28702354 | 122.4705642 | 1.82E-28 | 3.31E-27 |
| hsa-miR-222-3p | 1.122414495 | 5.707056939 | 120.5967908 | 4.68E-28 | 8.18E-27 |
| hsa-miR-500a-3p | -1.25708145 | 7.320071136 | 120.4427465 | 5.06E-28 | 8.51E-27 |
| hsa-miR-184 | 2.525685069 | 2.287766651 | 115.6672585 | 5.62E-27 | 9.11E-26 |
| hsa-miR-342-3p | -0.97450168 | 6.357688631 | 115.4087122 | 6.40E-27 | 1.00E-25 |
| hsa-miR-425-5p | -1.44881422 | 6.654933899 | 115.2870252 | 6.81E-27 | 1.03E-25 |
|  |  |  |  |  |  |

**Table S14: miRNAs that are differentially expressed between primary tumors and normal solid tissues using DEseq2 package**

| miRNA Name | baseMean | log2FoldChange | lfcSE | stat | pvalue | padj |
| --- | --- | --- | --- | --- | --- | --- |
| hsa-miR-93-5p | 3372.01886 | 1.827546405 | 0.101656084 | 17.97773761 | 2.91E-72 | 1.30E-69 |
| hsa-miR-200c-3p | 12083.1504 | 1.656504887 | 0.10625086 | 15.59050807 | 8.45E-55 | 1.88E-52 |
| hsa-miR-375 | 97423.9065 | 2.000137196 | 0.132990521 | 15.03969739 | 4.03E-51 | 5.98E-49 |
| hsa-miR-17-5p | 278.454744 | 1.55650991 | 0.106407181 | 14.62786524 | 1.87E-48 | 2.08E-46 |
| hsa-miR-182-5p | 18419.6 | 1.996323065 | 0.136625597 | 14.61163284 | 2.37E-48 | 2.11E-46 |
| hsa-miR-1251-5p | 3.41823106 | -2.58473037 | 0.177050644 | -14.59881934 | 2.86E-48 | 2.12E-46 |
| hsa-miR-148a-3p | 60104.5223 | 1.466762079 | 0.101992342 | 14.38110011 | 6.80E-47 | 4.32E-45 |
| hsa-miR-20a-5p | 243.448394 | 1.731278288 | 0.121363204 | 14.26526514 | 3.60E-46 | 2.00E-44 |
| hsa-miR-891a-5p | 7.74237955 | -2.853285335 | 0.200560779 | -14.22653693 | 6.27E-46 | 3.10E-44 |
| hsa-miR-25-3p | 6277.60161 | 1.134300298 | 0.082272023 | 13.78719354 | 3.04E-43 | 1.35E-41 |
| hsa-miR-92a-3p | 7314.91161 | 1.200409068 | 0.087159981 | 13.77247974 | 3.73E-43 | 1.51E-41 |
| hsa-miR-96-5p | 27.2899674 | 1.939749416 | 0.141242094 | 13.7335079 | 6.40E-43 | 2.35E-41 |
| hsa-miR-27b-3p | 1654.37548 | -0.943250001 | 0.068708389 | -13.72830917 | 6.87E-43 | 2.35E-41 |
| hsa-miR-204-5p | 24.3878737 | -1.819603814 | 0.138678369 | -13.12103556 | 2.50E-39 | 7.93E-38 |
| hsa-miR-889-3p | 13.5897485 | -1.196748008 | 0.091430185 | -13.0892003 | 3.80E-39 | 1.13E-37 |
| hsa-miR-153-5p | 32.6606 | 2.38335407 | 0.185524172 | 12.84659591 | 8.99E-38 | 2.50E-36 |
| hsa-miR-145-3p | 148.171414 | -1.151128333 | 0.092542529 | -12.43891149 | 1.61E-35 | 4.21E-34 |
| hsa-miR-23b-3p | 1549.1382 | -0.890905886 | 0.071692731 | -12.42672548 | 1.87E-35 | 4.63E-34 |
| hsa-miR-500a-3p | 158.794427 | 1.258362929 | 0.101798461 | 12.36131584 | 4.23E-35 | 9.91E-34 |
| hsa-miR-221-3p | 221.114751 | -1.450817877 | 0.117459805 | -12.35161153 | 4.77E-35 | 1.06E-33 |
| hsa-miR-183-5p | 5484.8664 | 1.708098802 | 0.139995134 | 12.20112984 | 3.07E-34 | 6.50E-33 |
| hsa-miR-19b-3p | 156.946898 | 1.374032733 | 0.113784954 | 12.07569798 | 1.42E-33 | 2.87E-32 |
| hsa-miR-425-5p | 99.9515808 | 1.440815899 | 0.119987781 | 12.0080219 | 3.22E-33 | 6.24E-32 |
| hsa-miR-652-3p | 11.3126493 | -1.144560594 | 0.096540752 | -11.85572487 | 2.01E-32 | 3.73E-31 |
| hsa-miR-342-3p | 80.8991007 | 0.97330278 | 0.083190413 | 11.69969883 | 1.28E-31 | 2.28E-30 |
| hsa-miR-136-3p | 13.2437757 | -1.413750638 | 0.120965825 | -11.68719043 | 1.48E-31 | 2.54E-30 |
| hsa-miR-99b-3p | 24.8619169 | -0.918331506 | 0.079022812 | -11.6210937 | 3.22E-31 | 5.31E-30 |
| hsa-miR-103a-3p | 14772.2009 | 0.881622425 | 0.076004999 | 11.59953205 | 4.14E-31 | 6.49E-30 |
| hsa-miR-141-3p | 938.791713 | 1.498197312 | 0.129179647 | 11.59778144 | 4.23E-31 | 6.49E-30 |
| hsa-miR-378c | 7.35090897 | -1.079533463 | 0.094042813 | -11.47917032 | 1.68E-30 | 2.49E-29 |

Table S15: Common miRNAs in serum and tissue

| Sources | Common miRNAs |
| --- | --- |
| Tissue & Serum | hsa-miR-133b, hsa-miR-31-3p, hsa-miR-27b-3p, hsa-miR-92a-3p, hsa-miR-23b-3p, hsa-miR-136-3p, hsa-miR-103a-3p, hsa-miR-191-5p,  hsa-miR-184, hsa-miR-23a-3p, hsa-miR-1307-3p, hsa-miR-24-3p, hsa-miR-423-3p, hsa-miR-629-5p, hsa-miR-151a-5p, hsa-miR-197-3p,  hsa-miR-17-3p, hsa-miR-26a-5p, hsa-miR-21-5p, hsa-miR-379-5p, hsa-miR-326, hsa-miR-615-3p, hsa-miR-331-3p, hsa-miR-323a-3p,  hsa-miR-320a, hsa-miR-425-3p, hsa-miR-411-5p, hsa-miR-15b-5p, hsa-miR-139-3p, hsa-miR-26b-5p, hsa-miR-542-5p, hsa-miR-29b-2-5p,  hsa-miR-107, hsa-miR-944, hsa-miR-1296-5p, hsa-miR-342-5p, hsa-miR-30b-3p, hsa-miR-5706, hsa-miR-4326, hsa-miR-451a, hsa-miR-133a-3p, hsa-miR-99a-5p, hsa-miR-409-5p, hsa-miR-22-3p, hsa-miR-125a-3p, hsa-miR-3130-5p, hsa-miR-654-5p, hsa-miR-125b-1-3p,  hsa-miR-185-3p, hsa-miR-106a-3p, hsa-miR-130a-3p, hsa-miR-28-5p, hsa-miR-140-3p, hsa-miR-24-1-5p, hsa-miR-194-3p, hsa-let-7d-3p,  hsa-miR-29b-1-5p, hsa-miR-1976, hsa-miR-128-1-5p, hsa-miR-574-3p, hsa-miR-195-3p, hsa-miR-629-3p, hsa-miR-151b, hsa-miR-769-3p, hsa-miR-3615, hsa-miR-320b, hsa-miR-183-3p |

**Table S16: Ranking of common miRNAs in serum, tissue, and urine using the RobustRankAggreg package**


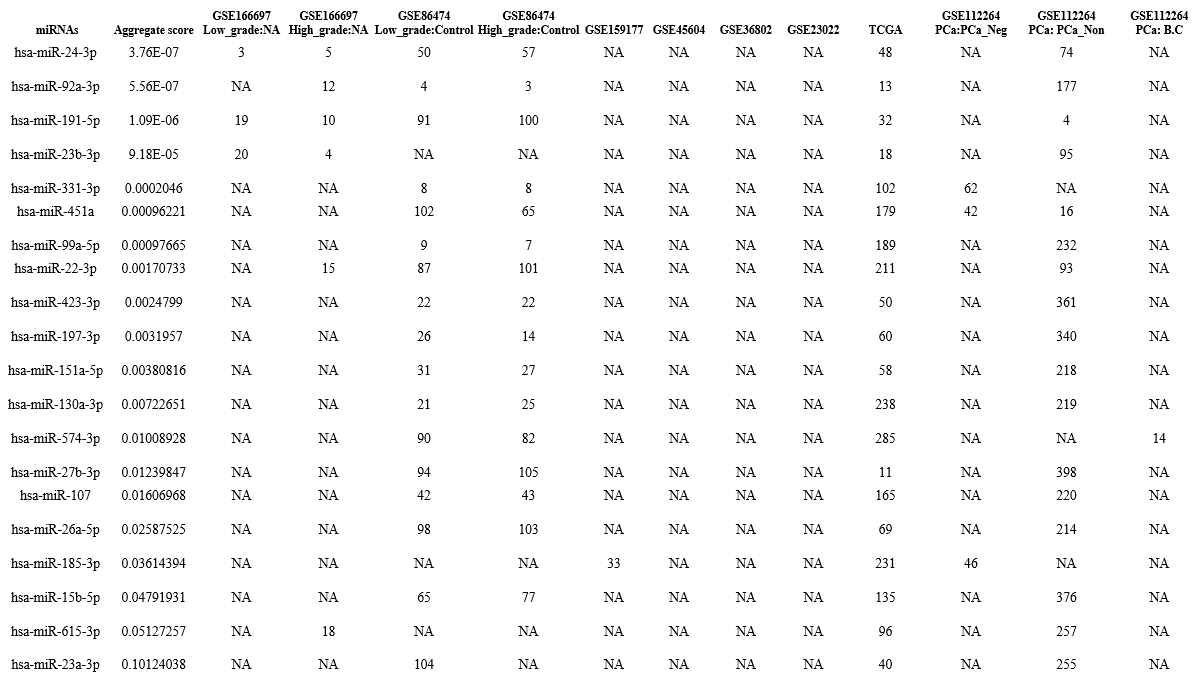


Table S17: The AUC values for double combinations of five selected miRNAs.


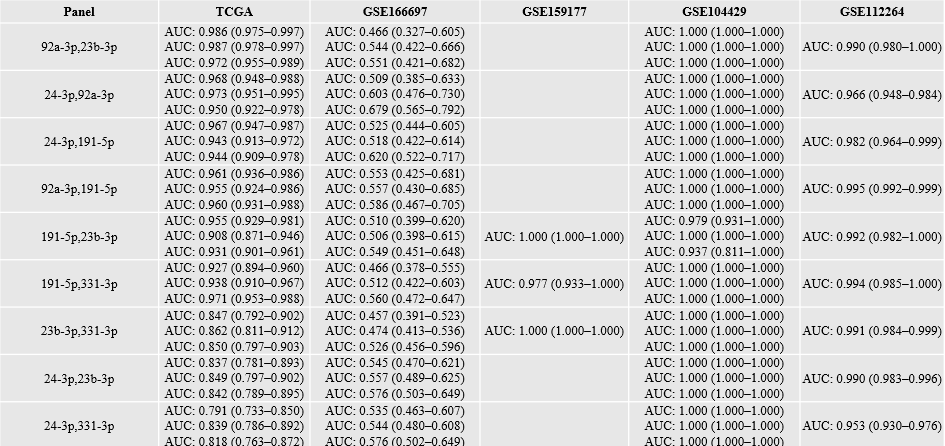


Table S18: The AUC values for triad combinations of five selected miRNAs.


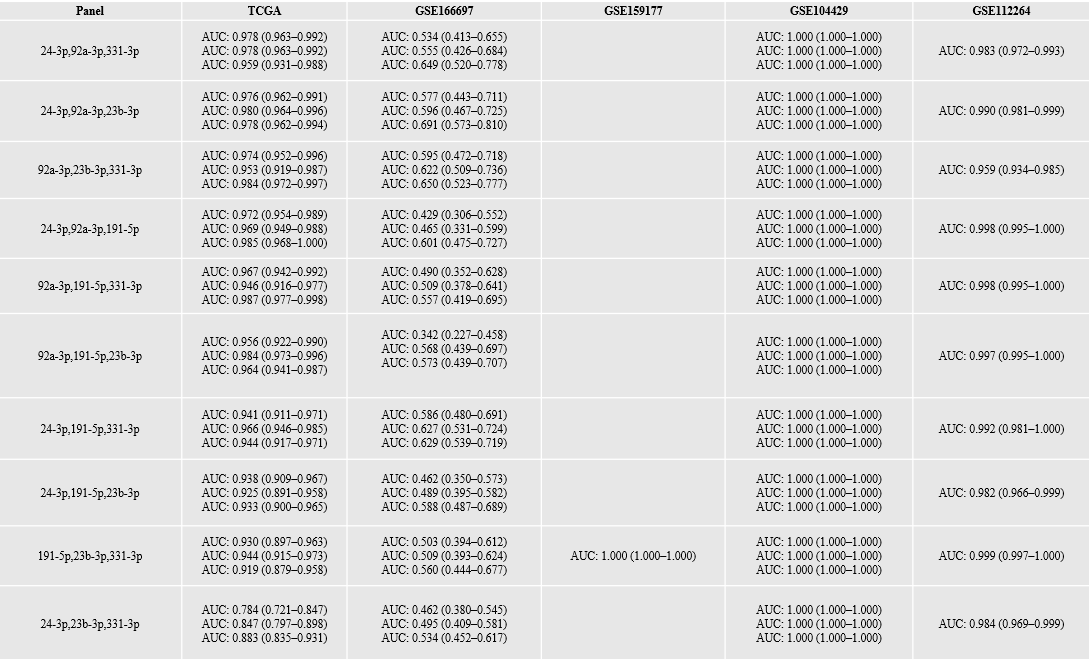

Supplement: Supplementary file 1 [file mmc1.docx]
